# Metabolomics and physio-chemical analyses of mulberry plants leaves response to manganese deficiency and toxicity reveal key metabolites and their pathways in manganese tolerance

**DOI:** 10.3389/fpls.2024.1349456

**Published:** 2024-06-05

**Authors:** Jianbin Li, Michael Ackah, Frank Kwarteng Amoako, Zipei Cui, LongWei Sun, Haonan Li, Victor Edem Tsigbey, Mengdi Zhao, Weiguo Zhao

**Affiliations:** ^1^Jiangsu Key Laboratory of Sericulture Biology and Biotechnology, School of Biotechnology, Jiangsu University of Science and Technology, Zhenjiang, China; ^2^Key Laboratory of Silkworm and Mulberry Genetic Improvement, Ministry of Agriculture and Rural Affairs, The Sericultural Research Institute, Chinese Academy of Agricultural Sciences, Zhenjiang, China; ^3^Institute of Plant Nutrition and Soil Science, Kiel University, Kiel, Germany; ^4^Department of Materials Science and Engineering, Suzhou University of Science and Technology, Suzhou, China

**Keywords:** mulberry, Manganese stress, physio-chemical, plant biomass, enzymatic activities, LC-MS, metabolomics, metabolic pathways

## Abstract

**Introduction:**

Manganese (Mn) plays a pivotal role in plant growth and development. Aside aiding in plant growth and development, Mn as heavy metal (HM) can be toxic in soil when applied in excess. *Morus alba* is an economically significant plant, capable of adapting to a range of environmental conditions and possessing the potential for phytoremediation of contaminated soil by HMs. The mechanism by which *M. alba* tolerates Mn stresses remains obscure.

**Methods:**

In this study, Mn concentrations comprising sufficiency (0.15 mM), higher regimes (1.5 mM and 3 mM), and deficiency (0 mM and 0.03 mM), were applied to *M. alba* in pot treatment for 21 days to understand *M. alba* Mn tolerance. Mn stress effects on the net photosynthetic rate (Pn), stomatal conductance (Gs), transpiration rate (Tr), intercellular CO_2_ concentration (Ci), chlorophyll content, plant morphological traits, enzymatic and non-enzymatic parameters were analyzed as well as metabolome signatures via non-targeted LC-MS technique.

**Results:**

Mn deficiency and toxicity decrease plant biomass, Pn, Ci, Gs, Tr, and chlorophyll content. Mn stresses induced a decline in the activities of catalase (CAT) and superoxide dismutase (SOD), while peroxidase (POD) activity, and leaf Mn content, increased. Soluble sugars, soluble proteins, malondialdehyde (MDA) and proline exhibited an elevation in Mn deficiency and toxicity concentrations. Metabolomic analysis indicates that Mn concentrations induced 1031 differentially expressed metabolites (DEMs), particularly amino acids, lipids, carbohydrates, benzene and derivatives and secondary metabolites. The DEMs are significantly enriched in alpha-linolenic acid metabolism, biosynthesis of unsaturated fatty acids, galactose metabolism, pantothenate and CoA biosynthesis, pentose phosphate pathway, carbon metabolism, etc.

**Discussion and conclusion:**

The upregulation of Galactinol, Myo-inositol, Jasmonic acid, L-aspartic acid, Coproporphyrin I, Trigonelline, Pantothenol, and Pantothenate and their significance in the metabolic pathways makes them Mn stress tolerance metabolites in *M. alba*. Our findings reveal the fundamental understanding of DEMs in *M. alba*’s response to Mn nutrition and the metabolic mechanisms involved, which may hold potential significance for the advancement of *M. alba* genetic improvement initiatives and phytoremediation programs.

## 1 Introduction

Soil acidification is becoming a serious environmental and ecological problem, posing a major threat to the functioning and structure of ecosystems in the world (Yin et al., 2023). Almost half of potential arable land belongs to acidic soil, which severely hampers agricultural production (Yin et al., 2023). One of the primary causes of soil acidification is the indiscriminate applications of chemical nutrients due to deficiencies. Manganese (Mn) is an essential trace element for plant growth and is widely regarded as the most distributed and abundant transition metal in nature (Neculita and Rosa, 2019; Bai et al., 2021). Functionally, Mn is known to be involved not only in growth and biomass production, but also in cascades of plant physiological and metabolic processes, viz., oxygenic photosynthesis, respiration, protein synthesis, fatty acids synthesis, and activating various enzymes (Wang et al., 2023). For example, Mn undergoes the oxygenic photosynthesis process by splitting water and oxygen gas (O_2_) in photosynthesis (Broadley et al., 2012). Mn not only functions directly in the formation, arrangement, and multiplication of chloroplasts, but also acts as an activator of enzymes in the photosystem II, Mn-containing superoxide dismutase (Mn-SOD) and oxalate oxidase (Broadley et al., 2012; Tewari et al., 2013; Wang et al., 2023). Additionally, Mn also serves as a co-factor in decarboxylases, RNA polymerases, and SOD, with other functions in the synthesis of secondary metabolites such as flavonoids, lignin, etc (Lidon et al., 2004; Li et al., 2019).

Although Mn contributes substantially to the growth and development of plants, but an optimal amount of it is required for normal growth and homeostasis (Wang et al., 2023). Indeed, this means that any Mn amount extremely below or above this optimum threshold renders it harmful and detrimental to the growth of the plant. For instance, Broadley et al. (2012) established a threshold of 10–20 µg Mn g^-1^ DM for Mn deficiency in older leaves, but the toxicity range reportedly varied among plant species, cultivars, and prevailing conditions. It has been reported that Mn toxicity widely increases in acidic soils when the pH is lower than 5.5 (Millaleo et al., 2013; Tewari et al., 2013). The excess Mn in soil does not only hinder growth and cause disruption to many physiological processes in plants, but also inhibits the acquisition and utilization of other nutrients, viz., iron, magnesium, calcium, and phosphorus, via complex formation and chelation (Wang et al., 2023). Considerable research findings have revealed that the growth and biomass accumulation of plants exposed to Mn toxicity are severely affected and reduced drastically compared with deficiency (Wang et al., 2023). For example, soybean plants treated with high Mn not only decreased the stem width, but also minimized the biomasses of shoots and roots relative to deficiency (Wang et al., 2023). Indeed, these developments warrant that the tendency of plants to cope with and adapt to Mn toxicity is quite imperative and requires urgent research attention.

Previous reports suggest that deficiency and excess application of nutrients are recognized as the principal causes of reactive oxygen species (ROS) production in plants, especially heavy metals like Mn (Kumar et al., 2007; Tewari et al., 2013). However, plants have evolved cascades of physiological changes and biochemical, metabolic, and transcriptional responses to adapt to and cope with these heavy transitional metals via a mechanism called phytoremediation (Mengdi et al., 2020). The rapid induction of antioxidants and antioxidant-related enzymes, including ascorbate peroxidase (APX), catalase (CAT), peroxidase (POD), and superoxide dismutase (SOD), has been reported by considerable studies to be the most commonly and frequently used adaptive strategy employed by plants during Mn toxicity (Ribera-Fonseca et al., 2013; Liu P. et al., 2019; Chang et al., 2020). For instance, Nazari et al. (2018) reported that progressively increasing Mn concentrations in Mentha aquatica plants simultaneously increased the contents of flavonoids, anthocyanins, malonaldehyde (MDA), hydrogen peroxide (H_2_O_2_), and activated antioxidant enzymes, such as SOD, CAT, and APX (Nazari et al., 2018). Apart from the antioxidant mechanisms, plants also cope with Mn toxicity by deploying or activating their tolerance mechanisms via regulation of Mn uptake (transport), translocation, and distribution within the plant cells (Wang et al., 2023). Some of these transporters (transport mechanisms) have been reported and include *AtNramp1*, and *AtNramp3*, *OsNramp1*, and *OsNramp5*, identified in *Arabidopsis* and rice, respectively (Socha and Guerinot, 2014; Chang et al., 2020). Additionally, plants exude organic acids that help to chelate Mn^2+^, which results in the formation of stable metal chelates that reduce Mn toxicity in plants (Kuang et al., 2022).

The use of omics approaches like metabolomics has recently helped in quantitative and qualitative analysis by elucidating the role of some unique metabolites used as an adaptive strategy by plants to cope with abiotic stresses (Mengdi et al., 2020). The inception of these omics approaches has led to the use of metabolomics for identifying and elucidating the various mechanistic pathways in plant metabolites in response to diverse stressors (Wang et al., 2023). Wang et al. (2023) identified a total of 300 differential metabolites when soybean plants were exposed to Mn toxicity. These metabolites were observed to have been directly involved in varied metabolic pathways in soybean, which consequently revealed that soybean exhibits varied adaptive mechanisms for coping with Mn toxicity. Furthermore, AgNPs improved respiration, inhibited photorespiration, and induced the synthesis of metabolites such as p-benzoquinone, lactulose, carbozole, citraconic acid, acetamilide, lactamide, and raffinose when cucumber plants were exposed to Ag^+^ (Zhang H. et al., 2018). Likewise, Wang et al. (2022) found that flavonoids were involved in Mn tolerance (Wang et al., 2022); however, the role of flavonoids in regulating Mn tolerance was reported as ambiguous and required further works.

Mulberry (*Morus alba* L.) is an excellent perennial woody plant with nutritional, medicinal, and ecological value (Ackah et al., 2021; Zhang Q. et al., 2023). *M. alba* plant has the potential to remediate contaminated soil including cadmium (Cd), making it a useful tool for phytoremediation (Lei et al., 2019). However, its growth and development have been hindered by nutrient deficiency and toxicity (Tewari et al., 2013; Jin et al., 2023; Zhang Q. et al., 2023). Notable among these nutrients are micronutrients such as iron (Fe), copper (Cu), zinc (Zn), boron (B), and Mn (Tewari et al., 2013; Zhang Q. et al., 2023). However, our comprehension of Mn deficiency and toxicity in *M. alba* has only been elucidated via physiological and biochemical means (Tewari et al., 2013). As it stands, the possible coordinating tolerance mechanisms elucidating *M. alba* plants response to deficiency and excess Mn using a combination of morpho-physiological, biochemical, and metabolomics approaches remain obscure. Hence, the aim of the current paper was to investigate the signatures of Mn deficiency and toxicity on growth, biomass, nutrient status, chlorophyll pigments, gas exchange, ROS scavenging antioxidants, and metabolite responses of *M. alba* plants exposed to Mn deficiency and toxicity. We hypothesized that an extreme Mn would cause disruption of the metabolic process, leading to higher metabolites production with lower contents in *M. alba* plants. The findings of this study provide vital information for further elucidating the Mn tolerance mechanisms in *M. alba* plants and sets a basis for genetically and biotechnologically improving Mn tolerance traits in *M. alba* plants.

## 2 Materials and methods

### 2.1 Plant material, growth conditions, and Mn stress treatments

The mulberry cultivar *M. alba* L. (Yu-711) was obtained from the National Mulberry GenBank at Jiangsu University of Science and Technology, Zhenjiang, Jiangsu, China. *M. alba* seedlings were raised from root stock for one and half month. The *M. alba* seedlings growth experiment was conducted within a controlled greenhouse environment, with seedlings cultivated in vermiculite. Methods outlined by Zhang Q. et al. (2023) were utilized in the cultivation of *M. alba* seedlings. To begin with the Mn treatments, fifteen Yu-711 seedlings in growth pot with vermiculite (without Mn nutrient supply) and having consistent growth conditions were chosen and divided into five groups. Each group contained three seedlings, with a seedling as replicate to ensure reproducible results. The seedlings were thereafter irrigated daily and supplemented with a solution of Murashige and Skoog (MS) medium (containing 4.37 g of MS media dissolved in 1000 mL of solution with a pH of 7.0) every three days for seven days. Subsequently, following the full growth of leaves, the plants underwent a 7-d treatment with deionized water. After this initial treatment, Mn (MnSO_4_) treatment was implemented based on the findings of a preliminary test, which established that 0.15 mM MnSO_4_ was the optimal concentration for promoting healthy growth of *M. alba* plants. This concentration was subsequently designated as the sufficiency (CK) group. Additionally, other concentrations were utilized for comparison, viz., T0 (0 mM) and T1 (0.03 mM) represented Mn -deficient groups, while T2 (1.5 mM) and T3 (3 mM) represented Mn -toxicity groups. Each group received 500 mL of MnSO_4_ nutrient solution containing the respective MnSO_4_ concentration, lasting for a period of 21 d. The application of the MnSO_4_ nutrient was done directly to the root in the soil (no foliar application). Twenty-one (21) d after treatments, leaves located at the 3–5 leaf position were collected from all groups. These leaves exhibited clear deficiency symptoms and were subsequently stored in a freezer at -80°C. For each experimental group, a combined sample of nine leaves was collected, with three leaves taken from each individual *M. alba* plant.

### 2.2 Determination of growth parameters and mineral analysis

The assessment of *M. alba* fresh and dry leaf weights was conducted in accordance with the methodology as outlined (Granado-Rodríguez et al., 2020). After the *M. alba* leaves were collected from the T0, T1, T2, T3, and CK groups, the fresh weight was measured using an electronic scale (BSA224S, Sartorius, Beijing, China). To determine the dry weight, *M. alba* leaves from each of the five groups were individually placed in separate beakers and subjected to the drying process using an electric blast drying oven (DHG-9140A, JingHong, Shanghai, China) set at 65°C for 48 h, during which time the leaf weight remained constant (indicating complete dryness). Each group consisted of three biological replicates, with each replicate containing three leaves. The study of Mn content in *M. alba* leaves and roots was conducted using an Inductively Coupled Plasma-Atomic Emission Spectroscopy/Mass Spectrometry (ICP-AES/OES/MS) apparatus (PerkinElmer, Waltham, MA, USA). This analysis was performed following the guidelines outlined in the National food safety standard for the analysis of multiple elements in food (GB 5009.268–2016). Using mixed sampling method, the roots and leaves of *M. alba* seedlings were sampled separately after treatment for 21 d. Samples were washed with deionized water and then dried with absorbent paper. Afterwards, the samples were oven-dried to constant weight at 65°C, and ground with mortar to a fine powder. A 0.2 g of dry sample was weighed in the PTFE (polyteflon) digestion tube, and 5 mL nitric acid was added to soak overnight. The digestion tubes were tightened and placed into microwave at 80°C for 2 h, and the temperature rose from 120°C for 2 h to 160°C. Then digest was transferred to a 25 mL volumetric flask and diluted with 100 mL H_2_O and were stored for measurement. A blank test was prepared. The Mn content was measured separately with Spectroscopy/Mass Spectrometry (ICP-AES/OES/MS). Each group consisted of three biological replicates, with each replicate containing three leaves and roots.

### 2.3 Assessment of photosynthetic parameters

To assess the net photosynthesis rate (Pn), stomatal conductance (Gs), intercellular CO_2_ (Ci), and transpiration rate (Tr), 3rd–5th leaves from each of the aforementioned groups were selected for measurement. On the 21 d of Mn stress, measurements were performed utilizing a PPSYSTEMS CIRAS-3 portable photosynthesizer from 9:00 to 11:00 a.m. The leaf temperature was at 25°C, the light intensity was established at 1200 μmol m^-2^ s^-1^, the relative humidity varied between 80% and 90%, and the atmospheric CO_2_ concentrations remained constant between 380–420 μmol mol^-1^. Each parameter was assessed with three biological replicates. Subsequently, analysis of variance and statistical significance (Tukey`s HSD, p < 0.05) was determined using R software v4.2. The stomatal opening of leaf samples was assessed using a QUANTA 250 FEG field emission scanning electron microscope (Thermo Fisher Scientific, Sunnyvale, CA, USA).

### 2.4 Determination of chlorophyll content

On the 21 d of treatment with varying MnSO_4_ concentrations, *M. alba* leaves were harvested from all experimental groups to assess their chlorophyll content. The chlorophyll content was ascertained utilizing the Plant Chlorophyll Content Kit (Trace Method) supplied by Keming Biotechnology Co., Ltd., Suzhou, China, in accordance with the instructions provided by the manufacturer. Following that, analysis of variance and statistical significance (Tukey`s HSD, p < 0.05) was determined using R software v4.2. Figures were plotted in Hiplot Pro (https://hiplot.com.cn/), a comprehensive web service for biomedical data analysis and visualization.

### 2.5 Determination of biochemical and physiological parameters

To determine biochemical and physiological indicators, *M. alba* leaves in all groups were collected on the 21 days of treatment after different concentrations of MnSO_4_ to evaluate the content of proline (PRO), soluble protein, soluble sugar, malondialdehyde (MDA). Also, enzymes such peroxidase (POD), superoxide dismutase (SOD), and catalase (CAT) activities were investigated. All the parameters were measured with detection Kit provided by Suzhou Keming Biotechnology Co., Ltd, Suzhou, China. The company’s Kit instructions were duly followed for the analysis and three biological replicates were used in each indicator. Data was processed and subsequently, analysis of variance and statistical significance (Tukey`s HSD, p < 0.05) was determined using R software v4.2. Figures were plotted in Hiplot Pro (https://hiplot.com.cn), a comprehensive web service for biomedical data analysis and visualization.

### 2.6 Correlation analyses between morphological, physiological and biochemical parameters

Pearson correlation heatmap for morphological, physiological and biochemical parameters measured in *M. alba* (Yu-711) grown in a pot (40 cm) experiment for 21 d under different levels of MnSO_4_ treatments was conducted using Corrplot tools in Hiplot Pro (https://hiplot.com.cn/). Correlation coefficient =1 or -1 was considered positive or negative correlation, respectively at p<0.05.

### 2.7 Sample preparation for metabolites extraction

All chemicals and solvents employed in the study were of high-performance liquid chromatography (HPLC) or analytical grade. A total of fifteen *M. alba* leaf samples were subjected to different Mn treatments, with three replicates for each treatment. These samples were then classified into five categories, namely T0, T1, CK, T2, and T3. To investigate the overall metabolomic patterns in the *M. alba* leaves, the metabolites of these samples were analyzed using an untargeted liquid chromatography-mass spectrometry (LC-MS) approach. The *M. alba* leaf samples (0.1 g) were promptly frozen in liquid nitrogen and subsequently pulverized into a fine powder using a mortar and pestle. The homogenate obtained was effectively combined with 1000 μL of a mixture consisting of methanol, acetonitrile, and water in a ratio of 2:2:1 (v/v/v). The solution was agitated using a vortex mixer, exposed to ultrasonication at low temperatures for a duration of 30 min, allowed to rest at -20°C for 10 min, and thereafter underwent centrifugation at 14000 g for 20 min at 4°C. Following collection, the supernatant was desiccated under vacuum. To reconstitute the sample for mass spectrometry analysis, 100 L of an acetonitrile/water solution (acetonitrile:water = 1:1, v/v) was introduced. The mixture was centrifuged at 14,000 g for 15 minutes at 4°C subsequent to vertexing, prior to being injected for LC-MS/MS analysis.

### 2.8 UHPLC-MS/MS analysis

Analysis was performed using an UHPLC (1290 Infinity LC, Agilent Technologies) coupled to a quadrupole time-of-flight (AB Sciex TripleTOF 6600). A column made of ACQUIY UPLC HSS T3 1.8 µm (Waters, Ireland) with a dimension of 2.1 mm × 100 mm was employed. Water containing 0.1% formic acid and acetonitrile containing 0.1% formic acid comprised the mobile phase in ESI positive mode; in contrast, acetonitrile comprised A=0.5 mM ammonium fluoride in water and B=acetonitrile in ESI negative mode. The gradient commenced at 1% B for 1.5 min, following which it was progressively elevated in a linear manner in 11.5 min until reaching 99% B. This was then maintained for an additional 3.5 min. Subsequently, it was decreased to 1% in 0.1 min, followed by a re-equilibration interval of 3.4 min. The flow rate of the gradients was 0.3 mL/min, while the column temperatures were maintained constant at 25°C. A 2 µL portion of each sample was introduced by injection. The spectra at both the first and second levels of the sample were acquired with the AB Triple TOF 6600 mass spectrometer. The source conditions for the ESI following the HILIC chromatographic separation are as follows: The ion source gas1 (Gas1) was set to a value of 60, while the ion source gas2 (Gas2) was also set to 60. The curtain gas (CUR) was maintained at a level of 30. The source temperature was maintained at 600°C, and the IonSpray Voltage Floating (ISVF) was set to ± 5500 V.

### 2.9 Data processing, quality control and metabolite identification

The software Compound Discoverer 3.1 (CD3.1, Thermo Fisher, USA) was employed to perform peak alignment, peak selection, and quantification for each metabolite utilizing the raw data acquired using ultra-high-performance liquid chromatography-tandem mass spectrometry (UHPLC-MS/MS). The key parameters were set as follows: a retention time tolerance of 0.2 min, an actual mass tolerance of 5 parts per million (ppm), a signal intensity tolerance of 30%, a signal-to-noise ratio of 3, and a minimum intensity threshold of 100,000. Following that, the intensities at the highest points were normalized in relation to the overall intensity of the spectrum. The molecular formula was subsequently deduced using the normalized data, taking into consideration additive ions, molecular ion peaks, and fragment ions. The peaks were subsequently cross-referenced with the mzCloud, mz Vault, and Mass List databases (https://www.mzcloud.org/), in order to obtain accurate qualitative and relative results. We implemented statistical analysis using Python (v 2.7.6), R (v3.4.3), and CentOS (v6.6). Metabolites were detected using both positive ion mode (POS) and negative ion mode (NEG) and distinct analyses were conducted on the positive and negative ion models. Additionally, QC samples were utilized in quality control inspections.

### 2.10 Hierarchical cluster analysis

On the data, hierarchical cluster analysis (HCA) was conducted, and the average linkage method was used to compute the resultant dendrogram. The R program pheatmap was utilized to hierarchically cluster the data after z-score normalization. The quantification of variation in metabolite composition and abundance among samples can be achieved through the utilization of correlation data. The correlation analysis was executed using the R package pheatmap, which also generated the heatmap.

### 2.11 PCA and OPLS-DA analysis

To conduct an initial examination of variations among distinct sample groups, the unsupervised dimensionality reduction technique known as principal component analysis (PCA) was employed on all samples. This analysis was performed using the R package models, (http://www.r-project.org/). Comparative groups were analyzed with partial least squares discriminant analysis (PLS-DA) utilizing the R package ropls. R package models were utilized to implement orthogonal projection to latent structures-discriminant analysis (OPLS-DA) on comparison groups. The OPLS-DA model underwent additional validation through the implementation of cross-validation and permutation tests. In order to conduct cross-validation, the dataset was divided into seven subsets, with each subset serving as a validation set. R^2^ represented the overall variance in the data matrix that the model accounted for. A predictive model was deemed acceptable if its Q^2^ value exceeded 0.4, and it was deemed satisfactory if its Q^2^ value exceeded 0.9. The 200-times random permutation of class labels by the permutation test yields a distribution of R^2^ and Q^2^ values.

### 2.12 Differential metabolite identification and KEGG annotations and enrichment analysis

To evaluate the metabolites that distinguished two groups most effectively, a variable importance in projection (VIP) score of the (O)PLS model was utilized. VIP threshold was set to 1. In filtering for differential metabolites, the t-test was additionally employed as a univariate analysis. Those with a p-value of *t-test*<0.05 and VIP≥1 was considered differential metabolites between two groups. For enrichment analysis and annotation, KEGG metabolic pathways were utilized to map metabolites. In contrast to the entire background, pathway enrichment analysis identified metabolic or signal transduction pathways that were significantly enriched in differential metabolites. The MetaboAnalyst module was also employed to assess pathway over-representation via Metabolic Set Enrichment Analysis (MSEA). The analysis was conducted using the Small Molecule Pathway Database (SMPDB) library and the overrepresentation analysis (ORA) Fisher’s exact test implemented in the R package MSEAp.

## 3 Results

### 3.1 Mulberry morphological response, biomass production and Mn content in tissues

**Figure 1:**
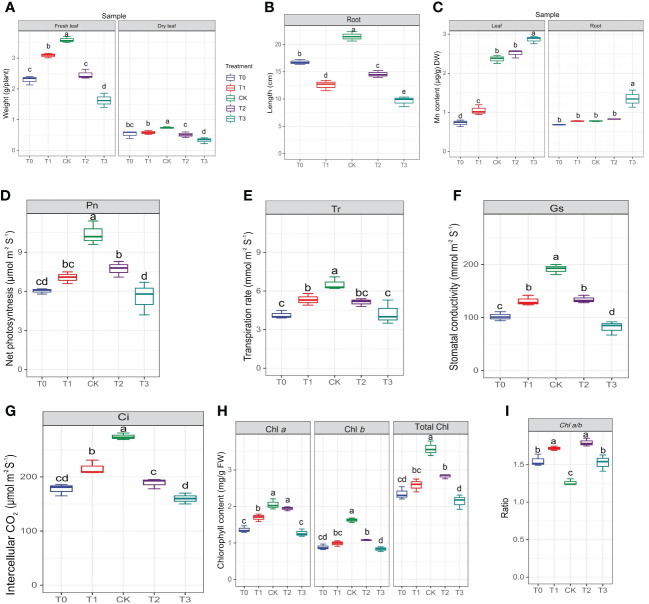
Morphological and determination of photosynthetic parameters of *M. alba* Yu-711 under different concentrations of Mn (MnSO_4_) treatment. **(A)** fresh leaf and dry leaf weight. **(B)** root length. **(C)** manganese content in leaves and root. **(D)** net photosynthetic rate (Pn). **(E)** transpiration rate (Tr). **(F)** stomatal conductance (Gs). **(G)** intercellular CO_2_ concentration (Ci). **(H)** Chlorophyll content. **(I)** Chlorophyll ratio. Values are means of three replicates of leaf samples. Different letters above the bar represent significant differences (Tukey’s HSD, p < 0.05). T0: 0 mM; T1: 0.03 mM; CK: 0.15 mM; T2: 1.5 mM; T3: 3 mM of MnSO_4_.

**Figure 2:**
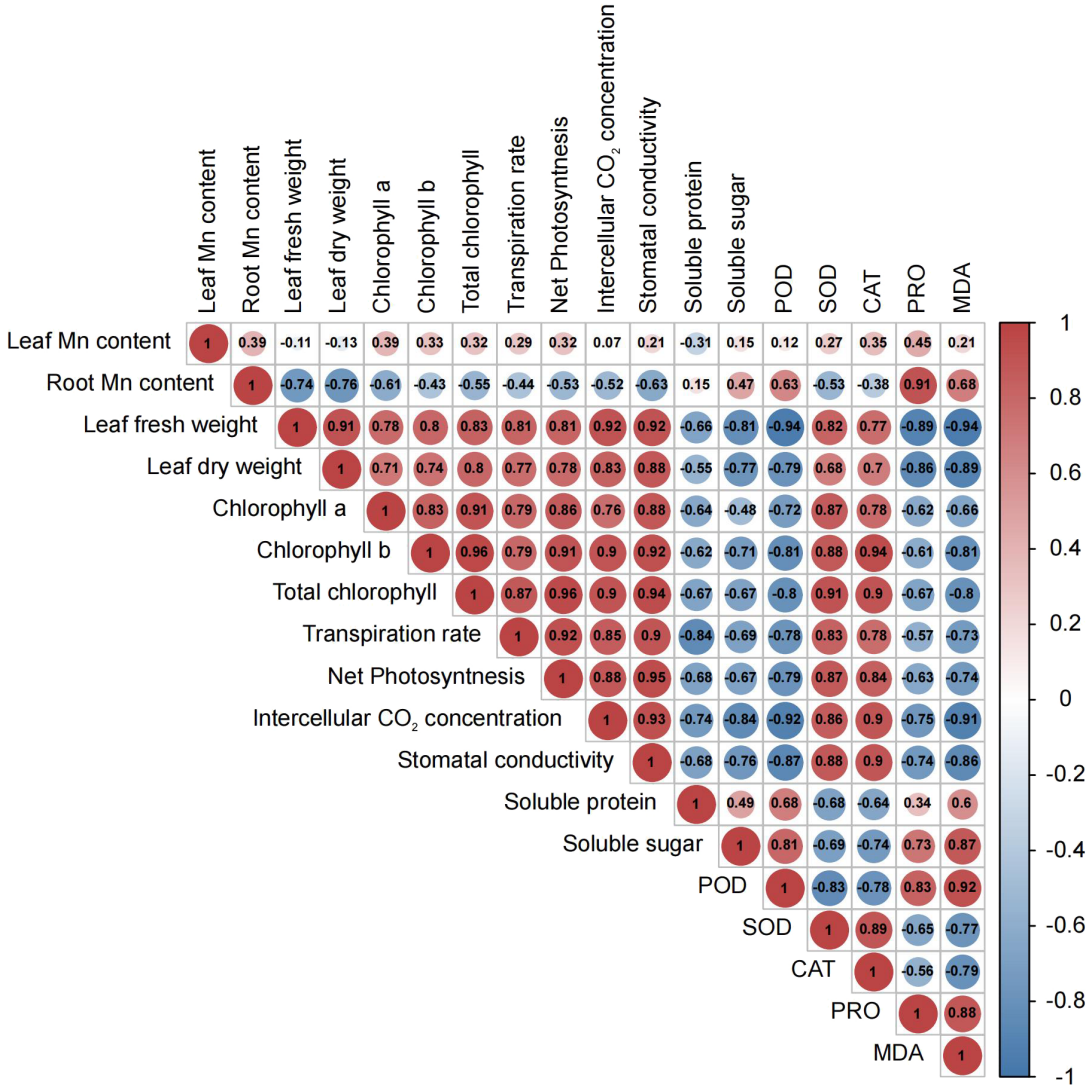
Pearson correlation heatmap for morphological, physiological, and biochemical parameters in *M. alba* (Yu-711) grown in a pot (40 cm) experiment for 21 days under different levels of MnSO_4_ treatments. The numbers in the circle represent the Pearson correlation coefficient. The red color indicates a positive correlation (positive value), and the blue color represents a negative correlation (negative value). From -1 to 1 represent the correlation (r) coefficient value. R= 0.5 or more is significant value either positive or negatively correlated.

Prior to conducting metabolome analysis and physiological and biochemical measurements, the morphology of leaves and roots was examined. The CK group displayed primary roots that were robust, as well as lateral roots that were well-developed (**Supplementary Figure S1**). The color of *M. alba* leaves changed from green to yellow, exhibiting symptoms of chlorosis, as observed in the T0, T1, T2 and T3 groups (**Supplementary Figure S1**). To determine how *M. alba* responded to Mn stresses, the morpho-physiological parameters of *M. alba* were examined. The findings indicate that there are statistically significant variations (p ≤0.05) across different Mn treatment concentrations (**Figure 1**). The findings suggest that an excess of Mn or a deficiency in Mn can hinder the development of the *M. alba* plant. Nevertheless, the treatment with adequate Mn (CK) exhibited remarkable growth in both fresh and dry biomasses as well as root length (**Figures 1A, B**). In comparison to *M. alba* plants treated with T0 (no Mn) and T3 (excess Mn), those treated with CK (sufficiency) exhibited fresh and dried weights increases of 36% and 54%, and 28% and 56%, respectively. This indicates that the fresh and dry weight of *M. alba* biomass is drastically reduced in *M. alba* toxicity as compared to the deficiency counterpart (**Figure 1A**). The accumulation of dry matter, on the other hand, was not substantially impacted by T0 or T1. Nevertheless, a notable distinction was observed in the accumulation of fresh and dry matter between treatments T2 and T3, indicating that treatment T3 (toxicity) exerted the most detrimental influence on biomass production. The levels of Mn in the leaves of *M. alba* plants exhibited significant variation, demonstrating a pattern consistent with normal distribution (**Figure 1C**). It was observed that the toxicity group, specifically T3, exhibited considerably elevated levels of Mn in both the leaves and roots compared to the deficiency and sufficiency groups. Surprisingly, the Mn content in the T2 group, as observed in both the leaves and roots, did not differ significantly from that of the sufficiency group (**Figure 1C**). An insignificant negative correlation (r = -0.11, -0.13) was identified between *M. alba* biomass (FW and DW) and the accumulated of Mn content in leaves (**Figure 2**).

### 3.2 Photosynthetic parameters and leaf pigments of *M. alba* in response to manganese

Considerable declines in net photosynthetic rate (Pn) were detected in *M. alba* plants that were deficient in Mn, as well as in groups that received low or high Mn supplies. Conversely, a rise in Pn was detected in the CK group (**Figure 1D**). Similar patterns were observed in the transpiration rate (Tr), stomatal conductance (Gs), and intercellular CO_2_ concentration (Ci), with CK demonstrating enhanced efficacy in comparison to the alternative interventions (**Figures 1E–G**). The findings of this study suggest that the physiological efficacy (growth) of *M. alba* plants in treatments T0, T1, T2, and T3 was significantly impaired as a result of response from Mn deficiency and toxicity to the photosynthetic apparatus. The weight measurements of fresh and dry leaves provide support for this assertion (**Figure 1A**). Nevertheless, it is important to highlight that the T0 and T3 regimens exhibited the most subpar performance across all assessments of the photosynthetic apparatus. It is noteworthy that while there were no substantial variations in Ci, Pn, and Tr between T0 and T3, significant shifts were detected in comparison to CK. Aside from Ci, T1 and T2 displayed a comparable pattern at these parameters. Consequently, the observed positive and statistically significant correlations (r = 0.92, 0.81, 0.81, and 0.92; FW) and (0.83, 0.78, and 0.88; DW) between plant biomass and gas exchange parameters (Tr, Pn, Gs, and Ci, respectively) could support the impact of Mn on photosynthetic efficiency (**Figure 2**).

As shown in **Figure 1H**, the availability of Mn significantly influences the content of chlorophyll (Chl) *a*, Chl *b*, and total Chl. In comparison to the sufficiency group (CK), Chl *a* level in T0 and T3 significantly decreased. However, there were no significant differences in Chl *a* level between T2 and CK. Chl *b* concentrations were significantly reduced across all treatments (T0–T3) in comparison to CK concentrations; which consequently led to the chlorotic and necrotic spot symptoms displayed in leaves of T0- and T3-induced plants (**Supplementary Figure S1A**; T0–T3). In *M. alba* plants supplied with sufficient Mn level (CK), the leaves exhibited normal growth without any leaf symptoms compared to other treatments (T0-T3). Nevertheless, there was no significant difference between T0 (deficiency) and T3 (toxicity); the same applies to T1 and T2 regarding Chl *b* contents. CK plants demonstrated the highest efficacy among the treatments evaluated in terms of total chlorophyll content, distinguishing itself considerably from both the toxicity and deficiency treatments. In contrast, T3 exhibited the lowest level of effectiveness. Conversely, T3 varied considerably from T0 and T2, whereas T1 and T2 did not differ significantly (**Figure 1H**). However, the CK exhibited the lowest Chl *a*/*b* ratio compared to other treatments (**Figure 1I**). The present result suggests that the adverse effects of excess Mn (toxicity) on chlorophyll or leaf pigments were more pronounced in comparison to the effects of shortage. The study revealed notable associations among chlorophyll content, biomass accumulation, and gas exchange parameters (**Figure 2**). As show in **Figure 2**, Chl *a*, *b*, and total Chl demonstrated a strong positive correlations with Ci (r = 0.76, 0.9, 0.9), Tr (r = 0.79, 0.79, 0.87), Gs (r = 0.88, 0.92, 0.94), Pn (r = 0.86, 0.91, 0.96), fresh biomass (r = 0.78, 0.8, 0.83), and dry biomass (r = 0.71, 0.74, 0.8), respectively (**Figure 2**).

**Figure 3:**
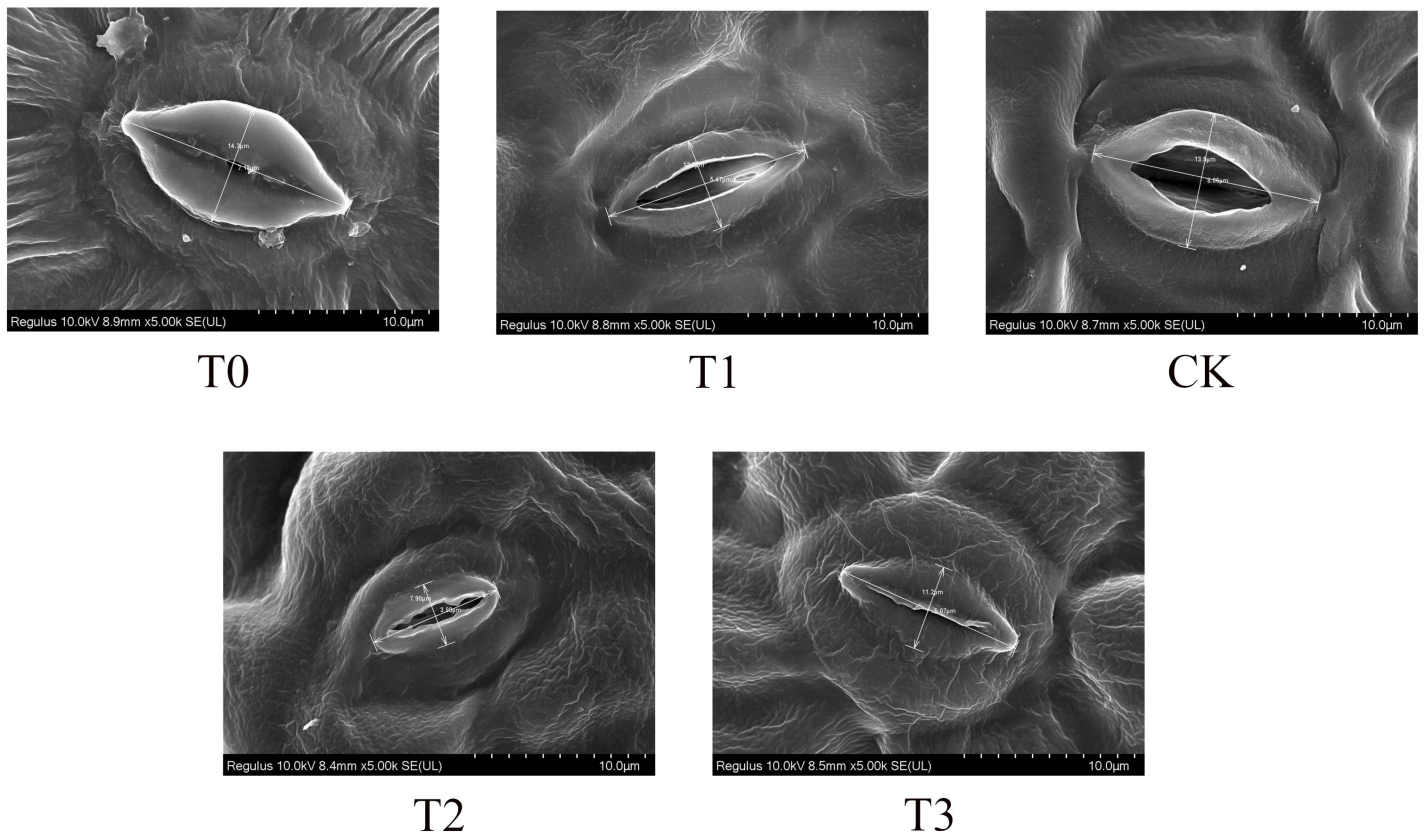
Stomatal conductance (Stomatal opening) of *M. alba* Yu-711 leaves under different concentrations of Mn treatment. T0: 0 mM MnSO_4_ treatment; T1: 0.03 mM MnSO_4_ treatment; CK: 0.15 mM MnSO_4_ treatment; T2: 1.5 mM MnSO_4_ treatment; T3: 3 mM MnSO_4_ treatment.

Field emission scanning electron microscopy was employed to analyze the adaxial leaf surface of *M. alba* plants (**Figure 3**). The stomatal conductance of *M. alba* plants was greatest in the CK group, as compared to the T1 and T2 groups. However, it was observed that groups T0 and T3 exhibited the least stomatal conductance, suggesting a propensity for stomatal closure. This observation highlights the profound influence that Mn deficiency and toxicity have on the conductance capacity of stomata.

### 3.3 Biochemical and enzymatic activities of *M. alba* plants in response to Mn supply

**Figure 4:**
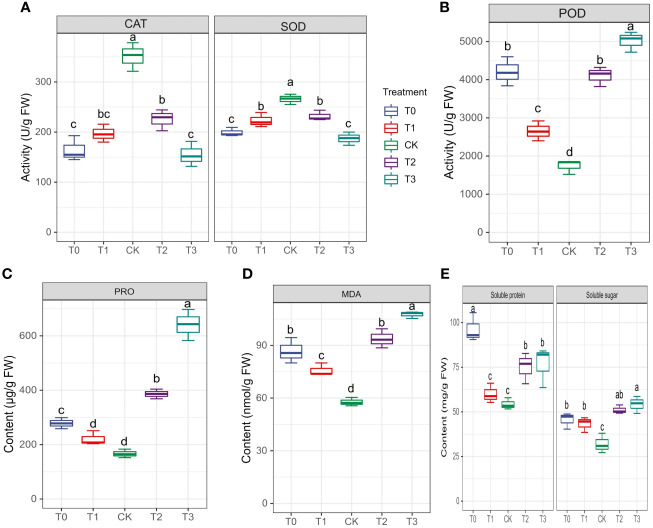
Physiological and biochemical indicators of *M. alba* Yu-711 under different concentrations of Mn (MnSO_4_) treatment. **(A)** CAT and SOD and activity. **(B)** POD activity. **(C)** PRO content. **(D)** MDA content. **(E)** soluble protein and soluble sugar content. Values are means of three replicates of leaf samples. Different letters above the bar represent significant differences (Tukey’s HSD, p < 0.05).

The activities of enzymes, particularly CAT, SOD and POD, were examined to evaluate *M. alba* response to Mn stress. Biochemical parameters such as PRO content, MDA content, soluble protein and sugar contents were evaluated (**Figures 4A–E**) The results of our investigation demonstrate that subjecting mulberries to an intense Mn stress initiated a series of biochemical alterations. Interestingly, in the measured enzyme activities, CAT and SOD activities in the T0 and T3 treatments significantly decreased, while CK exhibited an increase in activity (**Figure 4A**). Nonetheless, the activities of T1 and T2 were significantly higher than those of T0 and T3, indicating that T0 (zero Mn) and T3 (excessive Mn; toxicity) had a negative impact on CAT and SOD activities in *M. alba* plants. Conversely, POD activity shows a significant increase in the deficiency (T0 and T1) and toxicity (T2, and T3) treatments compared to CK (**Figure 4B**). Interestingly, there was no significant difference between T0 and T2, suggesting a similar response level to Mn deficiency and toxicity, with an elevated POD activity (**Figure 4B**). In comparison to the CK group, all treatment groups (T0-T3) exhibited elevated levels of proline (PRO) (**Figure 4C**). A similar pattern was observed for MDA content, with the highest levels recorded in the T0, T2 and T3 treatments. Meanwhile, CK observed the lowest (**Figure 4D**) as well soluble protein and sugar contents (**Figure 4E**), with the most significant increases being observed in T0 and T3. The results demonstrate that both Mn deficiency and toxicity led to notable elevations in the levels of soluble proteins, soluble sugars, proline (PRO), and malondialdehyde (MDA) in *M. alba* plants, as compared to the levels observed under sufficient Mn conditions. Correlation analysis shows that POD was negatively correlated with SOD (r=-0.83) and CAT (r=-0.75) but positively correlated with PRO (r=0.83) and MDA (r=0.92) (**Figure 2**). PRO correlated positively MDA (r=0.88), soluble sugar (r=0.73), soluble proteins (r=0.34). Soluble proteins and sugar were negatively correlated with all the photosynthetic parameters (**Figure 2**).

### 3.4 Metabolites profile in *M. alba* response to Mn supply and deficiency

Metabolomics in leaves were measure and not roots because *M. alba* leaves serves as the main food source for silkworm in sericulture production. Knowing metabolomics status in the leaves with regards to Mn nutrient is essential to produce quality leaves. A total of fifteen samples of *M. alba* leaves were subjected to different Mn treatments, with each treatment having three replicates. These samples were then categorized into five groups, namely T0, T1, CK, T2, and T3. To get insights into the overall metabolomic patterns in the *M. alba* leaves, the metabolites present in these samples were analyzed using an untargeted LC-MS approach. The aforementioned variables were subsequently examined among four distinct groups: CK-vs-T0, CK-vs-T1, CK-vs-T2, and CK-vs-T3. From the analysis, the results show that 2130 metabolites (1371 from positive-POS and 759 from negative-NEG ion mode) were obtained from the leaves sample treated with various Mn levels (**Supplementary Table S1**). Following normalization, the data obtained from the two ion modes were subjected to multivariate analysis using the R program, including PCA, PLS-DA, and OPLS-DA (**Supplementary Figure S2**). PCA score plot displayed significant metabolites variations after the different Mn treatments and deficiency. As shown in **Supplementary Figure S2A**, PCA of CK-vs-T0 exhibited 58.2% total variation, 67.4% in CK-vs-T1, 59.6% in CK-vs-T2, and 68.2% in CK-vs-T3 at the POS ion mode. Conversely, In the NEG ion mode, PCA displayed total variations of 59% in CK-vs-T0, 67.2% in CK-vs-T1, 60.1% in CK-vs-T2, and 67.3% in the CK-vs-T3 (**Supplementary Figure S2B**).

**Figure 5:**
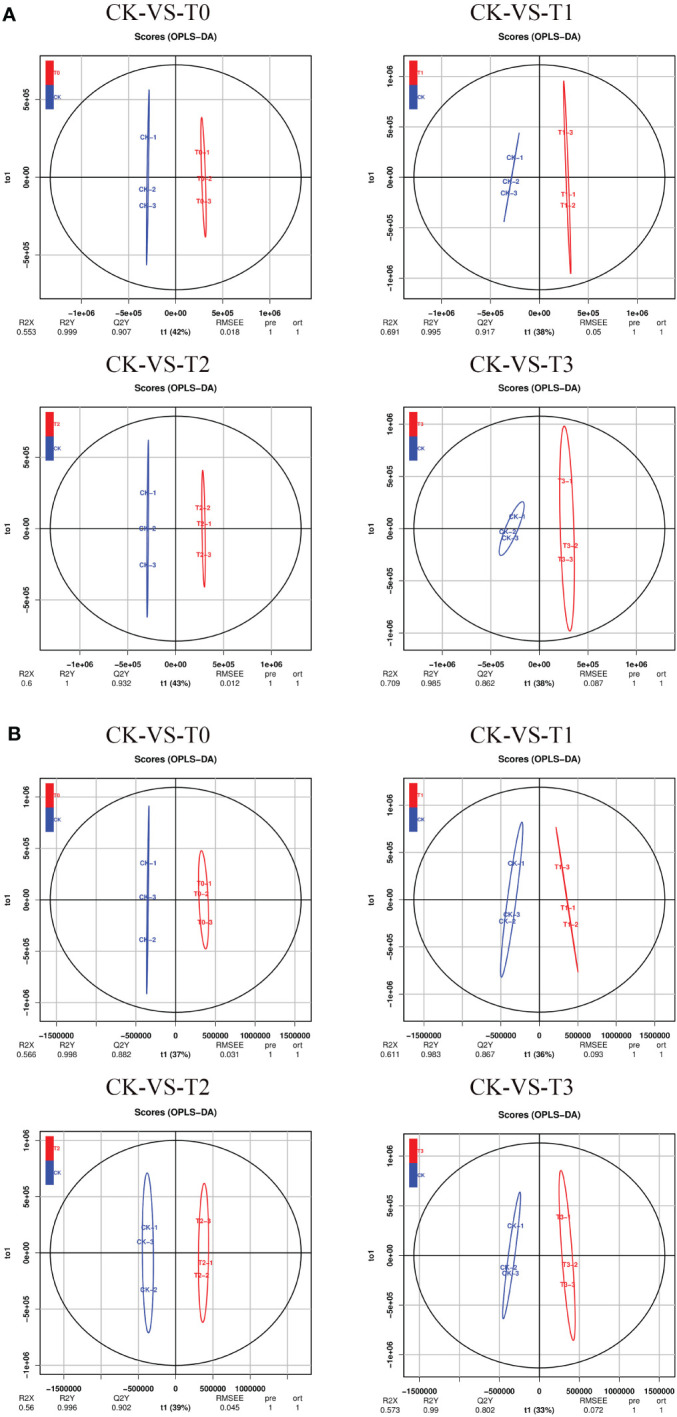
Orthogonal partial least-squares-discriminant analysis (OPLS-DA). **(A)** OPLS-DA score plot in POS ion mode. **(B)** OPLS-DA score plot in NEG ion mode. T0: 0 mM MnSO_4_ treatment; T1: 0.03 mM MnSO_4_ treatment; CK: 0.15 mM MnSO_4_ treatment; T2: 1.5 mM MnSO_4_ treatment; T3:3 mM MnSO_4_ treatment.

PLS-DA (**Supplementary Figure S3**) and OPLS-DA (**Figure 5**) analyses in the POS or NEG mode were performed on the difference between CK and the other Mn-treated groups, and similar classification results were obtained as in PCA. However, the OPLS-DA score analysis showed a clearer separation between the CK Mn-treated samples and deficient samples (T0 and T1) and Mn-toxicity samples (T2, T3). The OPLS-DA score plot explained that 42%, 38%, 43%, and 38% variations occurred between CK-vs-T0, CK-vs-T1, CK-vs-T2, and CK-vs-T3, respectively, at the POS ion mode (**Figure 5A**). The prediction satisfactory model parameters between CK-vs-T0, CK-vs-T1, CK-vs-T2, and CK-vs-T3 exhibited values ranging from Q2 = 0.907 (p-value; 0.018), 0.917(p-value; 0.05), 0.932 (p-value; 0.012) to 0.862 (p-value; 0.087), respectively (**Figure 5A**). Also, in the NEG ion mode, the OPLS-DA score plot reveals that CK-vs-T0, CK-vs-T1, CK-vs-T2, and CK-vs-T3 exhibited variations of 37%, 36%, 39%, and 33%, respectively (**Figure 5B**). The prediction model parameter of Q2 showed values of 0.882 (p-value; 0.031), 0.867 (p-value; 0.093), 0.902 (p-value; 0.045), and 0.802 (p-value; 0.072) for CK-vs-T0, CK-vs-T1, CK-vs-T2, and CK-vs-T3, respectively (**Figure 5B**). Comparable to the PCA, the OPLS-DA score plot displayed a discernible clustering pattern that indicated metabolomic differences between CK and the deficiency and the toxicity levels. This signifies the presence of a resilient and adequate discrimination model. The permutation testing of the OPLS-DA score distributions is depicted in **Supplementary Figure S4**.

### 3.5 Analysis of the differential metabolites in responses to Mn deficiency and toxicity

**Figure 6:**
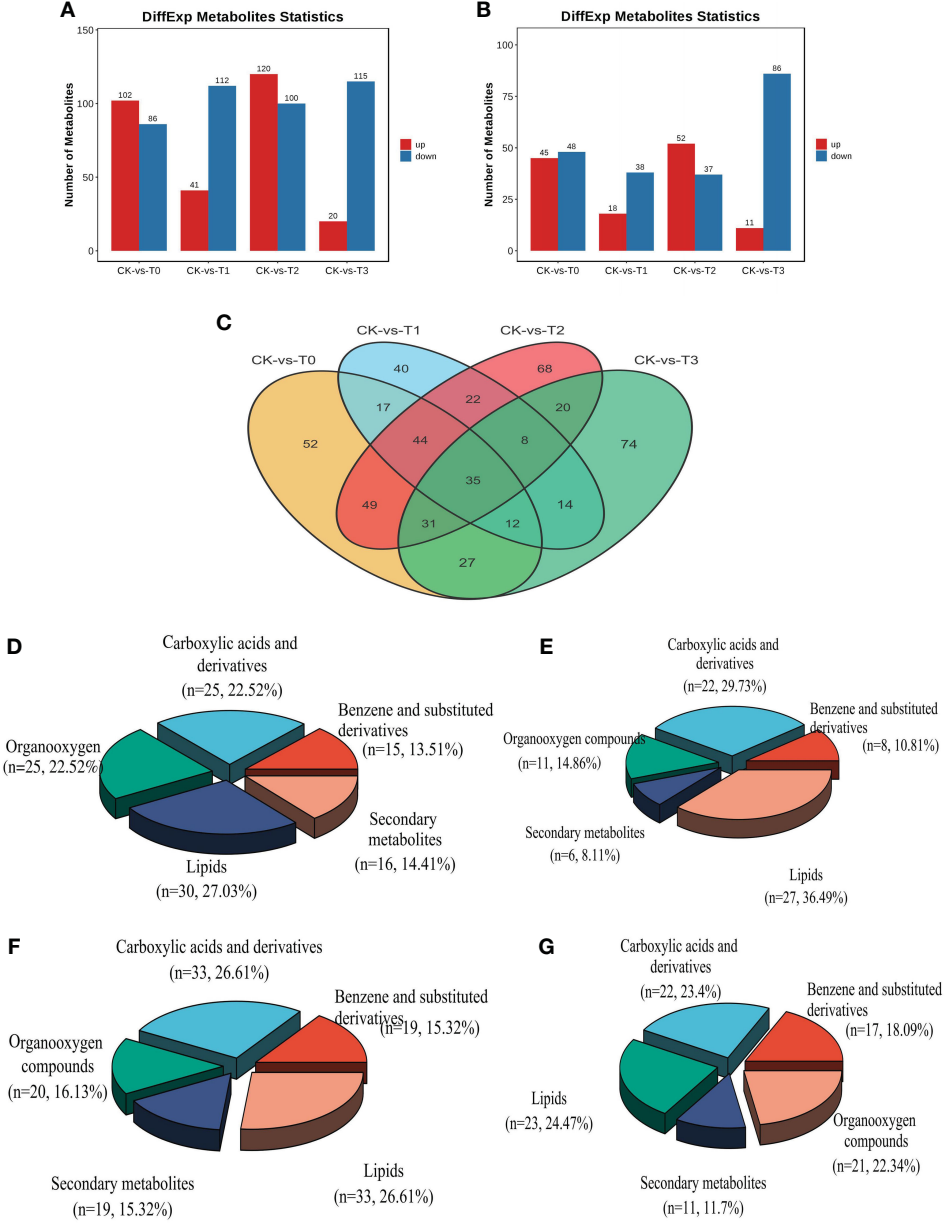
Statistics of the differential metabolites (DEMs) from group comparison. **(A)** number of differential metabolites from the POS ion mode **(B)** number of differential metabolites from the NEG ion mode. **(C)** DEMs distribution comparison in treatment groups. **(D–G)** DEMs classification in T0: 0 mM MnSO_4_ treatment; T1: 0.03 mM MnSO_4_ treatment; T2: 1.5 mM MnSO_4_ treatment; T3: 3 mM MnSO_4_ treatment, respectively. N = number of each metabolite in each class. The Red and blue color in the bar plot indicates upregulated and downregulated metabolites from group comparison.

Screening was performed on the differentially expressed metabolites (DEMs) in CK relative to the deficiency and toxicity samples, as determined by LC-MS ion modes. The DEMs underwent screening using VIP (Variable Importance in Projection) values. The OPLS-DA model employed a t-test with a significance level of 0.05 and a criterion of VIP≥1. The results of the DEMs analysis indicate that a total of 1031 DEMs were obtained from all the samples. In the POS ion mode, 696 (50.76%) DEMs exhibited between CK and T0-T3, encompassing 283 upregulated and 413 downregulated metabolites from a total of 1371 (**Supplementary Table S2**). Likewise, in the NEG ion mode, 335 (44.13%), consisting of 126 upregulated and 209 downregulated metabolites out of 759, showed DEMs between CK and T0-T3 (**Supplementary Table S3**). Furthermore, Mn deficiency groups (T0 and T1) obtained a total of 490 DEMs (284 down and 206 upregulated) in comparison with the CK (**Figures 6A, B**). On the hand, the Mn toxicity groups (T2 and T3) in comparison with the CK reveal a total of 541 DEMs comprising 338 down and 203 upregulation (**Figures 6A, B**). **Figure 6C** demonstrated that 35 DEMs were commonly expressed between all the treatments groups. Among the most significant DEMs in the deficiency groups include Cinchonine, Daurisoline, Solstitialin A 13-acetate, Rutin, Sebacic acid, all upregulated and Pirinixic acid, Entecavir, all downregulated (**Supplementary Tables S2** and **S3**). In the toxicity groups, the most significant DEMs included Terephthalic acid, 2,4,6-trichlorophenol, and Zearalenone, and were upregulated and Oleoyl ethylamide downregulated (**Supplementary Tables S2** and **S3**).

Analysis of the DEMs classification reveal that the metabolites were mostly classified into lipids, organooxygen, benzene and substituted derivatives, carboxylic acid derivatives, and secondary metabolites in both Mn deficiency group and toxicity (**Figures 6D–F**). In the Mn deficiency (T0; 0mM), lipids, mainly fatty acyl, steroids and their derivatives, prenol lipids, glycerophospholipids, etc., (**Supplementary Table S4**) accounted for 27.03% followed by carboxylic acid and derivatives (mainly amino acids; **Supplementary Table S5**) and organooxygen (mainly carbohydrates; **Supplementary Table S6**) accounting for 22.52% each, secondary metabolites (**Supplementary Table S7**) obtaining 14.41%, benzene and substituted derivatives (**Supplementary Table S8**) recording 13.51%. A similar trend of classification was observed in T2 (0.03 mM, **Figures 6D, E**). Additionally, in the Mn toxicity group (T3; 3 mM), lipids accounted for 24.27%, carboxylic acid and derivatives having 23.4%, organooxygen, benzene and substituted derivatives and secondary metabolites recording 22.34%, 18.09% and 11.7%, respectively (**Figures 6F, G**). Other classifications include Purine nucleosides, Pyrimidine nucleosides, Indoles and derivatives, lactones, along with some unclassified metabolites (**Supplementary Tables S2** and **S3**). The comparisons between the fold change [log2(FC)] and -log10 (p-value) of all the identified DEMs within the comparison groups were represented using volcano plots (**Supplementary Figure S5**). This allowed for a more comprehensive understanding of the upregulation and downregulation of these DEMs, based on their VIP value and p-value 0.05. To assess the impact of these DEMs and their contributions to the differentiation among samples, VIP values were analyzed. The results revealed that several DEMs had VIP values >1 in the OPLS-DA. **Supplementary Figure S6** presents the top 15 most significant metabolites (VIP≥1, p < 0.05) at the MS2 level that played a crucial role in the individual samples within the group comparisons. Some of these noteworthy metabolites include Reserpine, Desogestrel, Lpc18:2, Adenosine, Rhamnetin, Trans-3’-hydroxycotinine o-beta-d-glucuronide, and DL-asparagine (**Supplementary Figure S6**).

### 3.6 Hierarchical cluster analysis of DEMs

**Figure 7:**
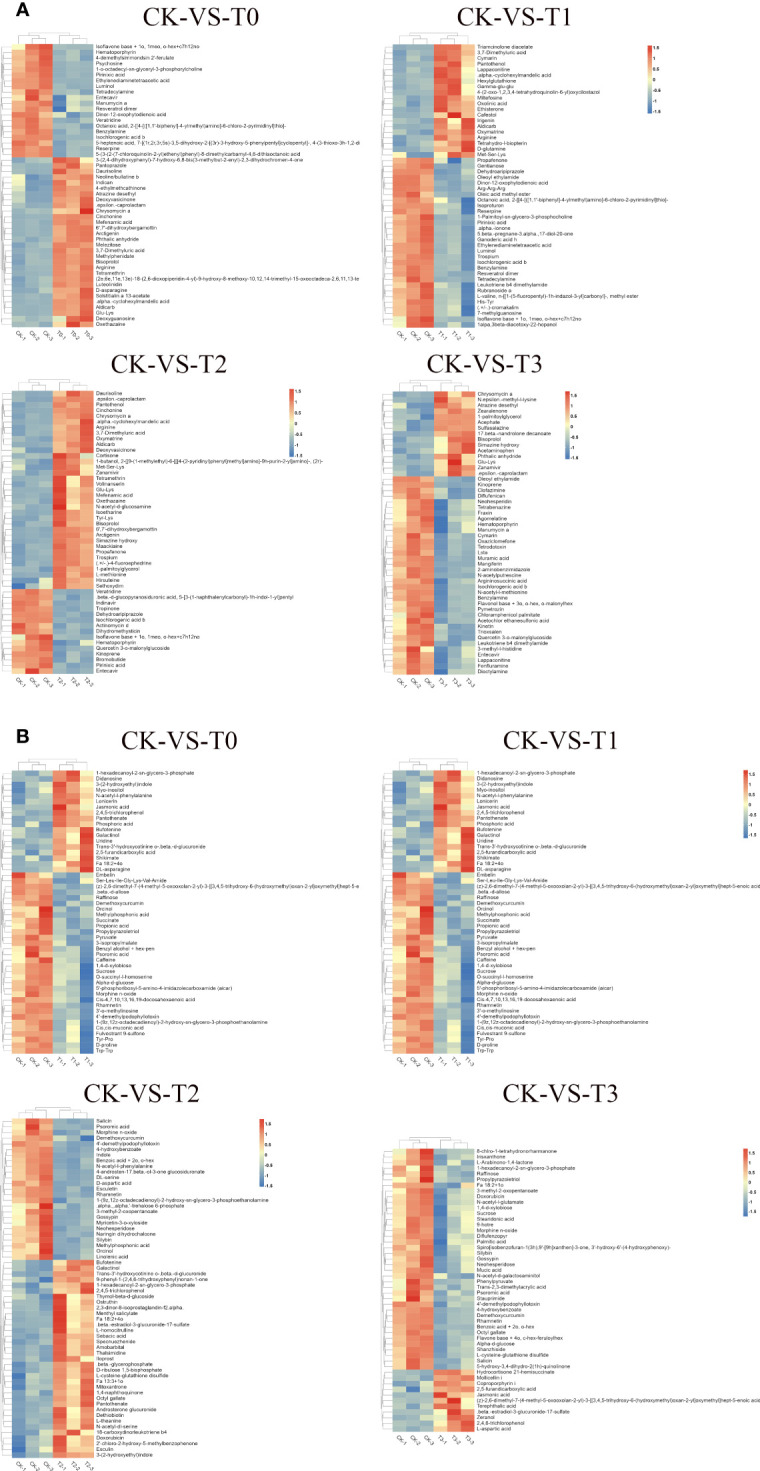
Hierarchical clustering analysis of differential metabolites. **(A)** pattern heat map of differential metabolites in POS ion mode. **(B)** pattern heat map of differential metabolites in NEG ion mode. The red color indicates high concentration, and the blue color indicates low concentration. T0: 0 mM MnSO_4_ treatment; T1: 0.03 mM MnSO_4_ treatment; CK: 0.15 mM MnSO_4_ treatment; T2:1.5 mM MnSO_4_ treatment; T3: 3 mM MnSO_4_ treatment.

Comparable expression patterns were identified among identical treatment groups by hierarchical cluster analysis (HCA), while noticeable variations were observed among distinct treatment groups (**Figure 7**). HCA (MS2 level) was conducted on the groups labeled as T0, T1, T2, and T3 to visually depict variations in metabolites with the CK groups. In the case of the deficiency group (T0), under the positive ion mode, higher concentrations of substances such as Cinchonine, Chrysomycin A, Arginine, D-asparagine, Daurisoline, and Solstitialin a 13-acetate, etc., were observed, while Isoflavone base + 1o, 1meo, o-hex+c7h12no, Pirinixic acid, Veratridine, and Entecavir, Hematoporphyrin, Psychosine, etc., exhibited lower concentrations in T0 (**Figure 7A**). In the negative ion mode, jasmonic acid, shikimate, 2,4,5-trichlorophenol, and Didanosine, Uridine, among others, demonstrated higher concentrations, whereas Raffinose, succinate, propionic acid, Pyruvate, D-proline, sucrose, caffeine, etc., displayed lower concentrations (**Figure 7B**). Furthermore, in the toxicity group (T3), when examined under the positive ion mode, higher concentrations were evident for Zearalenone, Chrysomycin A, and 17-beta-nandrolone decanoate, among others, while Oleoyl ethylamide, Entecavir, and Muramic acid, N-acetyl-l-methionine, Quercetin 3-o-malonylglucoside, Dicotylamine etc., displayed reduced concentrations (**Figure 7A**). Conversely, in the negative ion mode, Terephthalic acid, 2,4,6-trichlorophenol, and 2,5-furandicarboxylic acid, L-aspartic acid, zeranol etc., exhibited higher concentrations, while Phenylpyruvate, 8-chlro-1-tetrahydronorharmanone, and Fa 18:2 + 1o, sucrose, raffinose, N-acetyl-l-glutamate, palmitic acid etc., revealed lower concentrations (**Figure 7B**). The disparities regarding metabolites concentrations in T1, T2 with the CK are shown in **Figures 7A, B**.

### 3.7 Significant metabolic pathways induced in *M. alba* plant response to Mn deficiency and toxicity

**Figure 8:**
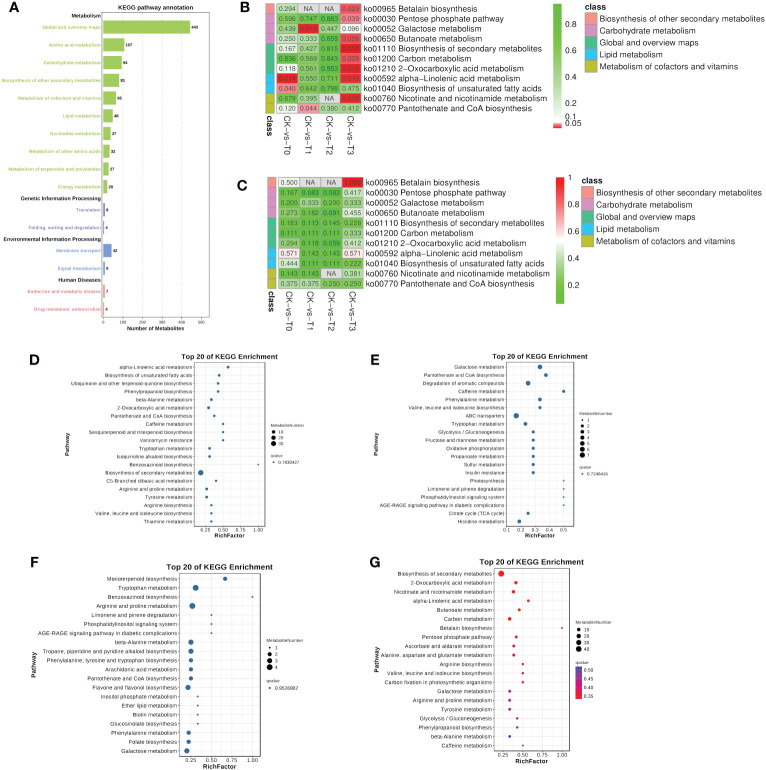
KEGG pathway enrichment analysis of the differential metabolites. **(A)** Statistical chart of metabolite KEGG annotations. The x-axis indicates the number of metabolites. **(B)** Significant enrichment pathways in each group comparison. **(C)** Pathway based on enrichment factor. Different colors represent classes of pathway. **(D–G)** Bubble plot showing the 20 KEGG pathways in T0: 0 mM; T1: 0.03 mM; CK:0.15 mM; T2: 1.5 mM; T3:3 mM of MnSO_4_ respectively. Bubble size indicates the number of metabolites. The bubble color from blue to red indicates an increase in significance (q-value).

**Figure 9:**
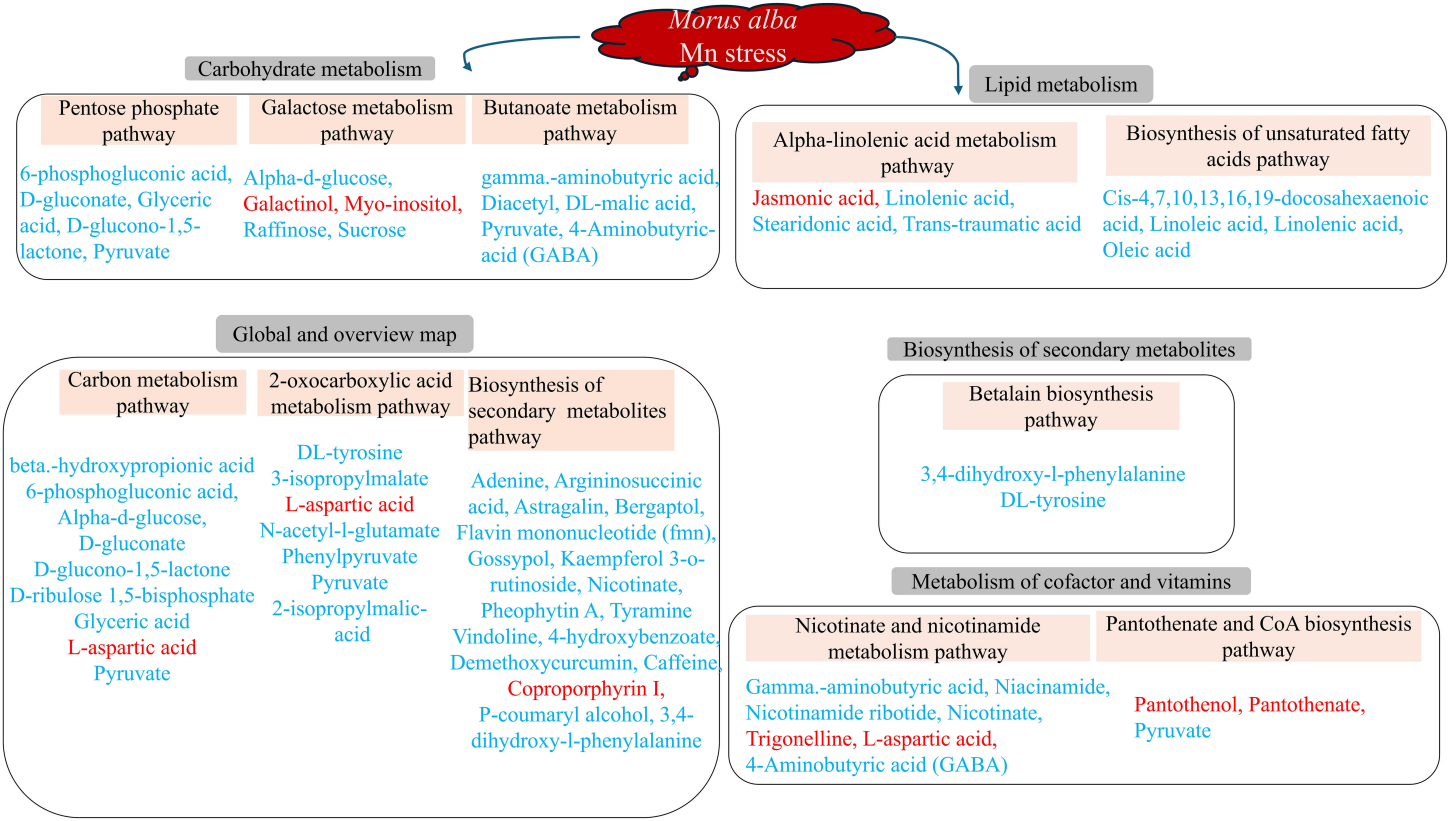
Molecular mechanisms of *M. alba* Mn deficiency and toxicity stress based on significant (p ≤ 0.05) KEGG pathways. Metabolites in red and blue colors are up- and down-regulated DEMs, respectively.

Metabolic pathways are very important in determining the most important biochemical metabolism and signal transduction pathways involved in metabolites, viz., the metabolism of carbohydrates, nucleosides, amino acids, etc., (**Figure 8A**) as reported (Kanehisa and Goto, 2000). The genetic information processing such as translation and folding, sorting and degradation were observed (**Figure 8A**). environmental information processing (membrane transport and signal transduction) and human disease (endocrine and metabolic disease and drug resistance: antimicrobial) were recorded (**Figure 8A**). Further analysis based on the most significantly enriched (p ≤ 0.05; lowest q-values) pathways reveal that four pathways were only significant in the Mn deficiency groups (T0, T1, **Figures 8D–G**). These include alpha-linolenic acid metabolism and biosynthesis of unsaturated fatty acids, galactose metabolism, and pantothenate and CoA biosynthesis etc., (**Figures 8D–G**). Metabolites such as jasmonic acid, linolenic acid, stearidonic acid, trans-traumatic acid, cis-4,7,10,13,16,19-docosahexaenoic acid, linoleic acid, and oleic acid were significantly enriched in the pathways (**Figure 9**). Surprisingly, none of the functional pathways were significant (FDR ≤ 0.05) in the T2 (**Figure 8**) in the toxicity. However, in T3, 8 significant pathways including pentose phosphate pathway, betalain biosynthesis, carbon metabolism, butanoate metabolism, nicotinate, and nicotinamide metabolism, etc., and betalain biosynthesis was highly enriched significantly (**Figures 8B, C**), suggesting that high Mn toxicity was detrimental to *M. alba* plants and hence triggered more metabolic pathways.

## 4 Discussion

### 4.1 Response of amino acids metabolites to Mn deficiency and toxicity in *M. alba*

Amino acids, aside from their role as building blocks of protein molecules, also serve as sources of energy, chemical messengers, and precursors for various metabolites, making them critical components of organism (Kawade et al., 2023). Each amino acid follows a typical metabolic pathway widely observed in plants, alongside some specific modifications (Gutiérrez-Preciado et al., 2010). Amino acids in plants exhibit metabolic plasticity during specific growth and developmental processes but are generally maintained at dynamic equilibrium levels (Hildebrandt et al., 2015). There is a growing body of research suggesting the potential roles of amino acids in regulating specific aspects of plant growth and development beyond their structural function (Hirai et al., 2023). In this study, several amino acids and their derivatives accumulated under Mn deficiency (0 mM and 0.03 mM), and Mn toxicity (1.5 mM and 3 mM) conditions (**Supplementary Table S5**). These amino acids include D-proline, 4-Aminobutyric acid (GABA), Arginine, L-aspartic acid, Trans-2-hydroxycinnamic acid, N-Acetylserotonin, DL-serine, N-acetyl-L-phenylalanine, N-acetyl-l-glutamate, Phenylalanine, and others. Verma et al. reported that proline is indispensable in plants’ response to various abiotic and biotic stressors (Verma et al., 2020). D-Proline exhibited relatively lower concentrations in the Mn deficiency and toxicity groups (Yu et al., 2023), however, most of the amino acids accumulated were relatively higher in concentration, which aligns well with the physiological outcomes such as soluble proteins (**Figure 4E**; **Supplementary Table S5**). The findings of this study indicate that the buildup of amino acids serves to regulate membrane homeostasis, facilitate osmotic adjustment, and improve stress tolerance (Li et al., 2021), as it was observed in PRO content in the Mn stressed plants compared to the sufficiency group (**Figure 4C**). Earlier reports suggested that *M. alba* plants subjected to other nutritional stress (boron and magnesium) accumulated higher PRO content (Jin et al., 2023; Zhang Q. et al., 2023). Our current result agrees with an earlier report that high Mn level in *Broussonetia papyrifera* (paper mulberry) increased PRO (Huang et al., 2019). The accumulation of D-proline in all treatment groups could again suggests a constitutive expression in response to Mn in *M. alba* plants.

Serine, as a metabolic factor, is a subject to intricate regulatory mechanisms. In the plastid serine biosynthesis (PSB) pathway, 3-phosphoglyceric acid (derived from the Calvin cycle or glycolysis cycle) is initially converted to 3-phosphohydroxypyruvate by the enzyme 3-phosphoglycerate dehydrogenase (PGDH) in the chloroplast. Subsequently, 3-phosphohydroxypyruvate is transformed into 3-phosphoserine by the enzyme 3-phosphoserine aminotransferase (PSAT). Finally, 3-phosphoserine is converted to serine by the enzyme 3-phosphoserine phosphatase (PSP) (Ros et al., 2014). Studies have reported that the reduced activity of PSP1 or PGDH1 also inhibits the root growth of *Arabidopsis thaliana* (Benstein et al., 2013). Moreover, serine serves as a primary precursor for tryptophan, which plays a crucial role in numerous biological processes such as plant growth, osmotic regulation, stomatal regulation, and acts as a precursor in the manufacture of plant growth hormones (Sadak and Ramadan, 2021). In our study, the level of DL-serine was reduced in the Mn deficiency (T0) and toxicity (T2) groups (**Supplementary Table S5**), highlighting that *M. alba* employed the down regulation mechanisms to adapt to Mn stress. This aligns with the observed phenotypic status of *M. alba* (**Supplementary Figure S1**). Interestingly, phenylalanine was exclusively expressed and significantly upregulated under Mn deficiency (T0 and T1) and not Mn toxicity (**Supplementary Table S5**), highlighting that phenylalanine could play significant role in elevating the nitrogen and amino acid metabolisms under Mn deficiency conditions to ensure effective growth and development of plants in response to diverse abiotic stresses.

The tricarboxylic acid (TCA) cycle holds significant importance as a metabolic route in plants, since it plays a vital role in supplying precursors for nitrogen metabolism and the production of amino acids. It exhibits a strong correlation with reactions to biotic stress and the maintenance of cellular homeostasis (Zhang Y. et al., 2018). In this study, aspartic acid was involved in the TCA cycle by participating in the aspartate-ketoglutarate transaminase reaction, combining with alpha-ketoglutarate. It showed higher expression levels in the Mn-toxicity (T2 and T3) *M. alba* leaves (**Supplementary Table S5**), suggesting its role in promoting the plant growth to resist stress conditions. Gamma-aminobutyric acid (GABA) is a non-protein amino acid initially discovered in potato tubers (Li et al., 2022). Its levels significantly increase in plants when subjected to various stressors such as drought, high salt, and high temperatures. GABA metabolism is predominantly facilitated via the GABA shunt, a brief pathway that circumvents two tricarboxylic acid (TCA) cycle reactions (Michaeli and Fromm, 2015). Additionally, it can be synthesized via polyamine degradation pathways (Zarei et al., 2016). In response to stress, increased GABA concentrations offer an alternative substrate for cellular activity enhancement (Renault et al., 2013). Additionally, it functions as a chemical safeguard against stress by functioning as a direct substrate for reactivating carbon and nitrogen metabolism subsequent to stress relief (Fait et al., 2011; Xu et al., 2022). In our study, aminobutyric acid exhibited lower concentrations in both Mn deficiency and toxicity groups (**Supplementary Table S5**), indicating that the Mn stresses affected protein synthesis for plant growth and development, which was observed in the plant biomass (**Figure 1A**), leaves and roots (**Supplementary Figure S1**).

### 4.2 Response of carbohydrates metabolites to Mn deficiency and toxicity

In plants, carbohydrates produced through photosynthesis are renowned for their crucial roles as a primary source of energy for plant growth and the stabilization of cell structures (Zulfiqar et al., 2020). Additionally, carbohydrates have been found to serve key functions as signaling molecules similar to hormones (Weiszmann et al., 2018). Sugars, particularly disaccharides like sucrose, as well as members of the raffinose and fructan families, also play a role in plant responses to ROS generated in non-biological stress conditions. Increasing evidence suggests that sugars possess antioxidant properties due to their ability to scavenge ROS (Keunen et al., 2013). The accumulation of specific carbohydrates in *M. alba* leaves was induced by Mn deficiency and toxicity in this investigation, which is consistent with the observed increase in soluble sugar content (**Figure 4E**; **Supplementary Table S6**). Under Mn toxicity conditions, the efficient operation of the Calvin cycle in CO_2_ fixation is hindered due to the influence of high NADP+. This results in a reduction in Ci (Santos et al., 2017), leading to an increase in Pn and water use efficiency (WUE). In this study, the Mn at 0.15 mM (CK) exhibited the highest gas exchange rates compared to the Mn deficiency and toxicity groups. The increase in Pn, Ci, Gs, and Tr can be attributed to the maximized CO_2_ fixation, which is reflected in the high Gs values and low Ci values. Optimal Mn level, in this regard, promoted stomatal opening, enabling the capture of higher concentrations of CO_2_ for use in the photosynthetic process. This is reflected in higher biomass, gas exchange rates, and controlled pigment levels in CK plants. As the severity of Mn toxicity and deficiency increased, both Pn, Ci, Gs and Tr in *M. alba* decreased (**Figures 1D–G**). It is speculated that the decline in Pn may be attributed to stomatal closure. Stomatal conductance, a crucial parameter, significantly impacts a plant’s photosynthetic and transpiration rates (Chutimanukul et al., 2022). In our study, we observed from the *M. alba* leaves that with the progression of Mn deficiency and toxicity, stomatal conductance gradually diminishes, ultimately approaching closure (**Figure 3**), which drastically impacted negatively on biomass.

The oxidative pentose phosphate pathway (OPPP) is widely present in higher plants, serving as the primary source of NADPH in eukaryotic cells and controlling metabolic intermediates involved in biosynthesis processes (Wang et al., 2017). Moreover, the OPPP is an essential pathway that maintains cellular redox balance under stress conditions in various plants (Landi et al., 2016). The 6-phosphogluconic acid, a key enzyme in the OPPP, plays a pivotal role in this process by decarboxylating CO_2_ molecules, reducing NADP^+^ to NADPH, and generating ribulose-5-phosphate (R5P) (de Freitas-Silva et al., 2017). In this study, an increase in 6-phosphogluconic acid content was observed in the Mn toxicity (T2 and T3) (**Supplementary Table S6**). Tian et al. reported an increase in the expression and activity levels of 6-phosphogluconic acid under cold stress in winter wheat (Tian et al., 2021). Beyond its role in responding to cold stress, 6-phosphogluconic acid also plays a significant role in response to heavy metal stress (Corpas et al., 2016). Galactinol synthase is a crucial precursor in the biosynthetic pathway of raffinose family oligosaccharides (RFO) (dos Santos and Vieira, 2020). Studies have shown that galactinol, myo-inositol and raffinose, in addition to their roles in carbon transport and storage, can also function in the signaling mechanisms of ROS accumulation, protecting plant cells from damage caused by ROS production (Nishizawa et al., 2008; Führs et al., 2011). In this study, an increase in a sugar alcohol, galactinol and myo-inositol contents were observed in the Mn deficiency groups (T0, T1) and the toxic group (T2) (**Supplementary Table S6**), suggesting its functional role in mitigating oxidative stress as a results of Mn deficiency and toxicity (Führs et al., 2011). Eastmond et al. (2015) revealed through labeling experiments that the catalysis of pyruvate to phosphoenolpyruvate by pyruvate orthophosphate dikinase (PPDK) is involved in the conversion of L-alanine (ALA) to sucrose, thus increasing soluble sugar content in plants and enhancing their resistance to abiotic stress (Eastmond et al., 2015). Our results reveal that both pyruvate and sucrose levels decreased in accumulation in Mn deficiency, with sucrose content observing downregulation patterns under toxicity (**Figure 9**, **Supplementary Table S6**). This phenomenon was observed in our physiological data, where soluble sugar content was higher in Mn stressed plants compared to the control (**Figure 4E**).

### 4.3 Response of antioxidants and secondary metabolites to Mn deficiency and toxicity

Excess Mn supply can produce oxidative bursts, leading to the generation of ROS such as superoxide radicals (O_2_^-^), hydrogen peroxide (H_2_O_2_), and hydroxyl radicals (OH^-^) (Boojar and Goodarzi, 2008). These ROS eventually instigate oxidative damage to membrane lipids, proteins, and nucleic acids (Boojar and Goodarzi, 2008). To mitigate the presence of ROS inside the plant system, enzymatic antioxidant systems play a crucial role. These systems involve the synthesis of certain enzymes, including superoxide dismutase (SOD), catalase (CAT), and peroxidase (POD) (Rahman et al., 2021; Shi et al., 2024), as well as the synthesis of non-enzymatic antioxidants like ascorbic acid, glutathione, phenolic compounds, and plant chelators are produced to protect plant cells. In our study, we observed a decrease in the activities of SOD and CAT in both Mn-deficient and toxic conditions compared to the Mn-sufficiency plants (**Figure 4A**), indicating the potential disruption of membrane systems and cellular homeostasis. It was reported earlier that Mn-deficiency increased total SOD and CAT activity, but excessive Mn supply reduced SOD and CAT activity (Tewari et al., 2013). This partly agrees with our results. On the other hand, increasing severity of Mn deficiency and toxicity in *M. alba* leaves increased the activity of POD but was significantly lower in the Mn sufficiency group (**Figure 4B**). According to Huang et al. (2019), Mn-defiecient and toxicity plants exhibited an increase - decrease -increase trend of SOD, CAT and POD with time in *B. papyrifera* response to Mn stresses (Huang et al., 2019). The results of SOD, CAT and POD activities in our study indicate that *M. alba* plants could use more of POD compared to SOD and CAT in preventing membrane damage in response to Mn stresses in ROS generation. Together, these maintain a balance between ROS formation and ROS detoxification, safeguarding cells from oxidative damage by ROS (Arora et al., 2002). Micić et al. (2013) reported that Mn toxicity induces oxidative damage in faba bean and increases the activity of antioxidant enzymes (Micić et al., 2013).

Plant secondary metabolites are compounds that play significant roles in the interaction between plants and abiotic stressors, distinct from the primary metabolites necessary for their basic survival. These secondary metabolites fulfill various critical functions in plants, including defense against pests and diseases, attraction of pollinators, interactions with other organisms, and responses to environmental stressors (Ashraf et al., 2018). Plant secondary metabolites encompass alkaloids, phenolic compounds, terpenoids, and carotenoids, typically not directly involved in growth and development but aiding plants in adapting to their surrounding ecological environment (Yang et al., 2018). Flavonoids, a subclass of polyphenolic compounds, are a type of secondary metabolite naturally occurring in many plants, widely distributed throughout the plant kingdom, primarily serving to protect plants from pests, UV radiation, and oxidative damage (Liu et al., 2021). Flavonoids act as antioxidants in plants, and their biosynthesis can be induced by environmental stressors such as metal ion exposure, contributing to the mitigation of oxidative damage and safeguarding plant tissues against the harmful effects of free radicals (Chen et al., 2018). We identified several DEMs with varied concentrations, which are directly implicated in flavonoid biosynthesis including metabolite classes such as Coumarins and derivatives, Isoflavonoids, Phenol ethers, Phenols etc., in *M. alba* leaves, highlighting the diverse class of metabolite altered in response to varied levels of Mn. Meanwhile, we found that most of these metabolites were downregulated (**Supplementary Table S7**), suggesting downregulation mechanisms employed my *M. alba* plants upon Mn imbalances. However, metabolite such as P-coumaryl alcohol, Rutin, Mefenamic acid, Astilbin and Tangeritin were uniquely expressed and significantly upregulated in T0 (**Supplementary Table S7**). This phenomenon indicates that these metabolites were markedly expressed and implicated in the biosynthesis of secondary metabolites and their precursors required by plants to curtail oxidative damage under Mn deficiency. For instance, P-coumaryl alcohol has been revealed to exhibit certain biological properties such as antioxidant-related properties, with the capability to neutralize free radicals, contributing to the reinforcement and protection of plant cell walls (Yu et al., 2009). Luteolinidin is a member of the cyanidin family and, like other cyanidins, possesses antioxidant properties that can assist plants in combating abiotic stress (Wang et al., 2018). We found an increase (upregulation) in the concentration of Luteolinidin in *M. alba* leaves of the T0 group, which served to alleviate oxidative damage caused by Mn deficiency. Intriguingly, Acetaminophen was identified to be significantly elevated under extreme stress (**Supplementary Table S7**). Acetaminophen was exclusively altered expressed under Mn deficiency (T0) and toxicity (T3), indicating that Acetaminophen is a crucial metabolite in *M. alba* plants in response to Mn stress via mitigating of ROS and other oxidative stresses. Tramadol is a synthetic opioid analgesic compound and reported to have induce antioxidant production in mammals (Mohammadnejad et al., 2021). It has been revealed to scavenge free radicals, lessens oxidative stress, and protect cells from damage triggered by ROS in plants. For example, tramadol (TRD)-treated barley plants observed remarkable inductions in guaiacol peroxidase, catalase and glutathione S-transferase in roots compared to the controls after 24 days of treatment (Khalaf et al., 2023). We observed up- and downregulation of tramadol in Mn deficiency (T0) and toxicity (T3), respectively (**Supplementary Table S7**), signifying differential and contrasting mechanisms employed by *M. alba*, mechanisms potentially assisting in alleviating oxidative stress in plants by scavenging free radicals and reducing cellular damage in response to Mn deficiency and toxicity. The differential regulation of tramadol expression could suggest bifunctional roles in curbing stresses in *M. alba* plants. This is consistent with the POD and MDA activities (**Figure 2**), which have been reported to increase by the induction of tramadol (Mohammadnejad et al., 2021). Similarly, 6’,7’-dihydroxybergamottin, belonging to Coumarins and derivatives class of secondary metabolites, also observed differential mechanistic regulation in this study. The up- and downregulation of 6’,7’-dihydroxybergamottin under extreme Mn stresses highlights its direct implications in scavenging ROS and oxidative damage via an antioxidant-related properties in plants (**Supplementary Table S7**). Führs et al. (2012) reported higher metabolites in the phenylpropanoid pathway in the leaf of the Mn-sensitive cv. TVu-91 plants, which is in accordance with our current findings. It is quite plausible to hypothesized that *M. alba* plants, under Mn imbalances trigger and employ differential regulation mechanisms to cope with lack of Mn and tolerate Mn toxicity via detoxifying excess ROS production. Our results further suggest that the differential expression of metabolites under specific Mn conditions indicate non-constitutive expression and confirming the metabolic reprogramming mechanisms induced by Mn imbalance in *M. alba* plants. The increase in the synthesis of phenolic compounds under heavy metal stress has been reported to help in phytoremediation due to their high tendency to chelate metals due to the presence of the hydroxyl and carboxyl groups that bind to metal ions such as iron, copper, Mn etc., (Singh et al., 2015). Hence, observing higher phenolic compounds under Mn stress, especially under toxicity conditions, could ensure chelation of Mn under Mn-toxicity conditions. A condition that can reduce the uptake of excess Mn to alleviate cell damage and abiotic stress in plants.

### 4.4 Response of lipids to Mn deficiency and toxicity

Lipids are a class of biologically organic compounds, encompassing various molecular types including fats, phospholipids, triglycerides, and lipid-soluble vitamins (Ackah et al., 2021). They constitute a major component of plant membranes, playing a pivotal role in maintaining cell structure (Liu X. et al., 2019), energy storage, signal transduction, antioxidant defense, osmotic regulation, and resistance to external stressors (Guo et al., 2019). Different Mn levels as well as deficiency in this study altered the expression of several key differential lipids in *M. alba* leaves, primarily comprising fatty acyls (FA), prenol lipids (Pl), glycerophospholipids (GPL), glycerolipids (GL), and sphingolipids (**Supplementary Table S4**). In the FA class, comparing Mn deficiency (T0) group to the toxicity (T2 and T3) groups, lipids such as jasmonic acid, sebacic acid, pantothenol, 3-isopropylmalate, dethiobiotin were upregulated in (T0), whereas sebacic acid, pantothenol, and dethiobiotin were only in T2. However, jasmonic acid was the only upregulated metabolite in the T3. This suggests that these compounds, responding to Mn deficiency and toxicity in *M. alba* may be the mechanism *M. alba* use to adjust its metabolic process for adaptation and survival during nutritional stress. Jasmonic acid (JA), a major plant hormone, exists in multiple active forms, including methyl jasmonate (MeJA) and cis-jasmone. These active metabolites serve as signaling molecules, activating plant defense genes when exposed to biotic and abiotic stressors (Griffiths, 2020). Thus, the upregulation of jasmonic acid in both the deficiency (T0) and higher toxicity (T3) further suggest that jasmonic may be *M. alba’s* nutritional stress tolerance molecule. Pantothenol, which is a precursor of pantothenic acid (vitamin B5), is a vital molecule for the growth and advancement of plants. It assumes a pivotal function in diverse biochemical pathways, comprising synthesis of coenzyme A, which is indispensable for energy metabolism and the formation of lipids, proteins, and other significant molecules (Gao et al., 2022). Thus, the upregulation of pantothenol in both Mn deficiency and toxicity (T2) suggest that pantothenol play a critical role in *M. alba* response to Mn stress by supporting essential metabolic processes and helping the plant to cope with the challenges posed by Mn deficiency or imbalance. In the prenol lipids (Pl), metabolites including costunolide and vitamin k1 2,3-epoxide were exclusive to T0 and were upregulated. However, ginkgolide C was exclusively expressed in Mn-treated samples and were upregulated in T1 and T2 but downregulated in T3 (**Supplementary Table S4**). This clearly shows that *M. alba* may recruit costunolide and vitamin k1 2,3-epoxide to deal with Mn deficiency stress and uses mechanism involving ginkgolide c in regulating its metabolism in response to Mn imbalances (T1-T3). Ginkgolide C, a compound found in *Ginkgo biloba* leaves, has been studied for its potential role in plant stress responses by providing antioxidants stress response, and ginkgolides, including ginkgolide C, are believed to contribute to the tree’s stress tolerance (Yan et al., 2021). Therefore, expression of ginkgolide C only in the Mn-treated samples may provide antioxidant properties that can help *M. alba* plant to mitigate the effects of Mn imbalances by scavenging ROS and reducing oxidative damage (Yan et al., 2021). Glycerophospholipids represent the most abundant class of phospholipids in organisms and serve as a crucial component of the bilayer membrane structure in cell membranes. They are involved in membrane recognition and signal transduction related to protein interactions (Zhang J. et al., 2023). In our study, 1-stearoyl-2-hydroxy-sn-glycero-3-phosphoethanolamine was upregulated and exclusive to T0 group whereas 1-Palmitoyl-sn-glycero-3-phosphocholine, and beta.-glycerophosphate were downregulated and exclusive to Mn-treated plants T1 and T2 or T3 (**Supplementary Table S7**). The changes in expression levels of these compounds in response to various levels of Mn treatments further highlights the complex metabolic response in *M. alba* nutrient stress response (Jin et al., 2023; Zhang Q. et al., 2023).

### 4.5 Mechanisms of *M. alba* metabolism in response to Mn tolerance

Understanding the metabolic mechanism regulating plant stress response is critical to investigating plant physiological and molecular mechanism in adapting to its environment. Metabolites including 6-phosphogluconic acid, D-gluconate, D-glucono-1,5-lactone, glyceric acid, pyruvate, gamma aminobutyric acid, diacetyl, 4-aminobutyric acid (GABA), Dl-malic acid, D-ribulose 1,5-bisphosphate, and L-aspartic acid were involved in the high toxicity-treated samples (**Figure 9**; **Supplementary Table S9**). *M. alba* response to Mn deficiency and toxicity induced some very important metabolic pathways in response to Mn tolerance (**Figure 9**). Specifically, high Mn toxicity (T3; 3 mM) induced several metabolic pathways compared to the deficiency groups. *M. alba* response to Mn deficiency and toxicity recruited down regulated metabolites in the significant pathways indicating that *M. alba* may use these mechanisms to mitigate Mn stress effects to maintain its growth and development. Some of the DEMs were involved in more than one of the significant metabolic pathways. Among them include pyruvate, 4-aminobutyric acid (GABA), D-glucono-1,5-lactone, DL-tyrosine, 6-phosphogluconic acid, nicotinate, etc., which were all down regulated (**Figure 9**). Only few of the metabolites involved in the significant pathways were upregulated and they include L-aspartic acid, galactinol, myo-inositol, coproporphyrin I, jasmonic acid, trigonelline, pantothenol and pantothenate. This further suggest that the upregulation of these metabolites may be crucial tolerance compounds involving *M. alba* metabolism reprogramming against heavy metal nutritional stress.

## 5 Conclusions

The response and tolerance mechanisms of the *M. alba* (Yu-711) leaves to Mn deficiency and toxicity were elucidated in this study. Morphological observations indicate a visible impact on *M. alba* growth under Mn treatments, particularly in the significant inhibition of lateral root numbers and length caused by Mn toxicity. In summary, our results indicate that *M. alba* leaves respond to varying Mn supply levels by regulating antioxidant enzyme activity, accumulating osmolytes, and modulating the expression of metabolites and altering metabolic pathways to counteract the effects. The upregulation of Galactinol, Myo-inositol, Jasmonic acid, L-aspartic acid, Coproporphyrin I, Trigonelline, Pantothenol, and Pantothenate and their significance in the metabolic pathways makes them Mn stress tolerance metabolites in *M. alba.* This study shed lights on the *M. alba’s* plant ability to cope with various Mn stresses, providing basis for further studies and insights.

## Data availability statement

The data presented in the study are deposited in the MetaboLights repository, accession number MTBLS10211.

## Author contributions

JL: Data curation, Formal analysis, Writing – original draft, Writing – review & editing, Investigation, Methodology. MA: Data curation, Formal analysis, Investigation, Methodology, Writing – original draft, Writing – review & editing, Conceptualization. FA: Data curation, Formal analysis, Writing – original draft, Writing – review & editing. ZC: Resources, Visualization, Writing – review & editing. LS: Resources, Software, Writing – review & editing. HL: Resources, Visualization, Writing – review & editing. VT: Resources, Software, Writing – review & editing. MZ: Conceptualization, Funding acquisition, Formal analysis, Writing – review & editing. WZ: Conceptualization, Funding acquisition, Project administration, Supervision, Writing – review & editing.
