# Supplementary material for: Metabolomics and physio-chemical analyses of mulberry plants leaves response to manganese deficiency and toxicity reveal key metabolites and their pathways in manganese tolerance
Source: Front Plant Sci. 2024 Jun 5;15:1349456. doi: 10.3389/fpls.2024.1349456 (PMC11192020; doi:10.3389/fpls.2024.1349456)
Supplement: Supplementary file 1 [file DataSheet_1.docx]

**Supplementary Figures**

**Figure legends**

**Figure S1:** Morphology of mulberry Yu-711 under different concentrations of Mn (MnSO_4_) treatments. (A) morphology of leaves. (B) morphology of roots. T0: 0 mM MnSO_4_ treatment; T1: 0.03 mM MnSO_4_ treatment; CK: 0.15 mM MnSO_4_ treatment; T2: 1.5 mM MnSO_4_ treatment; T3: 3 mM MnSO_4_ treatment.

**Figure S2** Principal component analysis (PCA). (A) PCA score plot in POS ion mode. (B) PCA score plot in NEG ion mode. T0: 0 mM MnSO_4_ treatment; T1: 0.03 mM MnSO_4_ treatment; CK: 0.15 mM MnSO_4_ treatment; T2: 1.5 mM MnSO4 treatment; T3: 3 mM MnSO4 treatment.

**Figure S3** Partial least-squares discriminant analysis (PLS-DA). (A) PLS-DA score plot in POS ion mode. (B) PLS-DA score plot in NEG ion mode. T0: 0 mM MnSO4 treatment; T1: 0.03 mM MnSO_4_ treatment; CK: 0.15 mM MnSO4 treatment; T2: 1.5 mM MnSO_4_ treatment; T3: 3 mM MnSO_4_ treatment.

**Figure S4**. Permutation test evaluating the accuracy of (O)PLS models. (A) in POS ion mode. (B) in NEG ion mode. The two rightmost points (x=1.0) are R2 and Q2 of the original model, and all the points on the left are the R2 and Q2 of the model after Y displacement; if all the blue Q2 points from left to left are lower than the original blue Q2 points on the far right, or the regression line of Q2 points is less than or equal to 0 at the intersection of ordinates, it means that the model prediction results are reliable. T0: 0 mM MnSO4 treatment; T1: 0.03 mM MnSO4 treatment; CK: 0.15 mM MnSO4 treatment; T2: 1.5 mM MnSO4 treatment; T3: 3 mM MnSO4 treatment.

**Figure S5.** Volcano plot of differential metabolites. (A) differential metabolites in POS ion mode. (B) differential metabolites in NEG ion mode. Red color indicates upregulation, and the green color indicates downregulation. T0: 0 mM MnSO4 treatment; T1: 0.03 mM MnSO4 treatment; CK: 0.15 mM MnSO4 treatment; T2: 1.5 mM MnSO4 treatment; T3: 3 mM MnSO4 treatment.

**Figure S6.** The Top 15 differential metabolites (MS2 level) and the VIP values. (A) VIP values of differential metabolites at the POS ion mode. (B) VIP values of differential metabolites at the NEG ion mode. The x-axis represents the VIP value, the left side of the y-axis represents the differential metabolite name, and the right side represents the differential metabolite concentration (the red color represents high concentration, green color represents low concentration).T0:0mM; T1:0.03 mM; CK: 0.15 mM;T2: 1.5 mM; T3:3 mM of MnSO_4_.

**Figure S1**
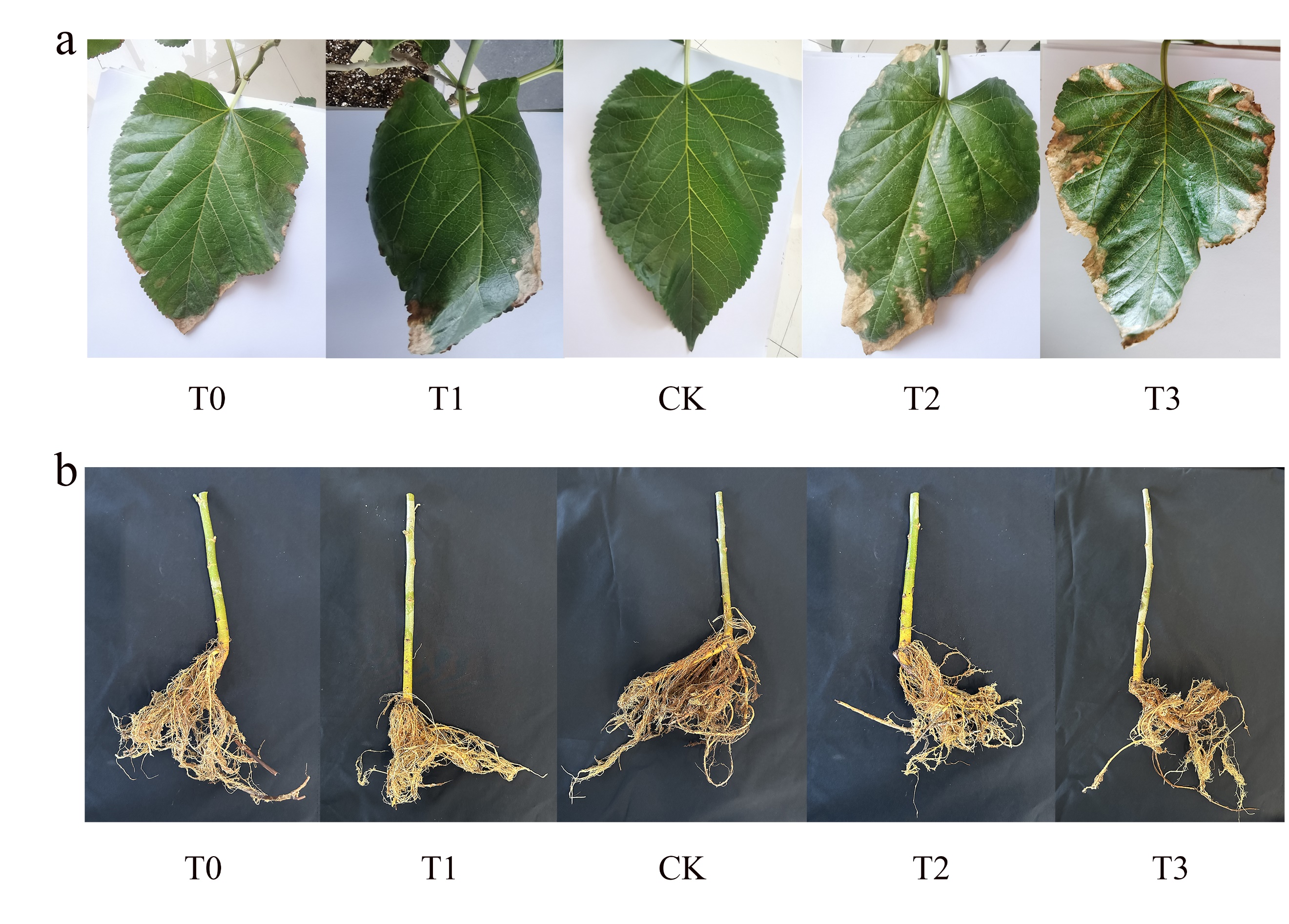


**Figure S2**


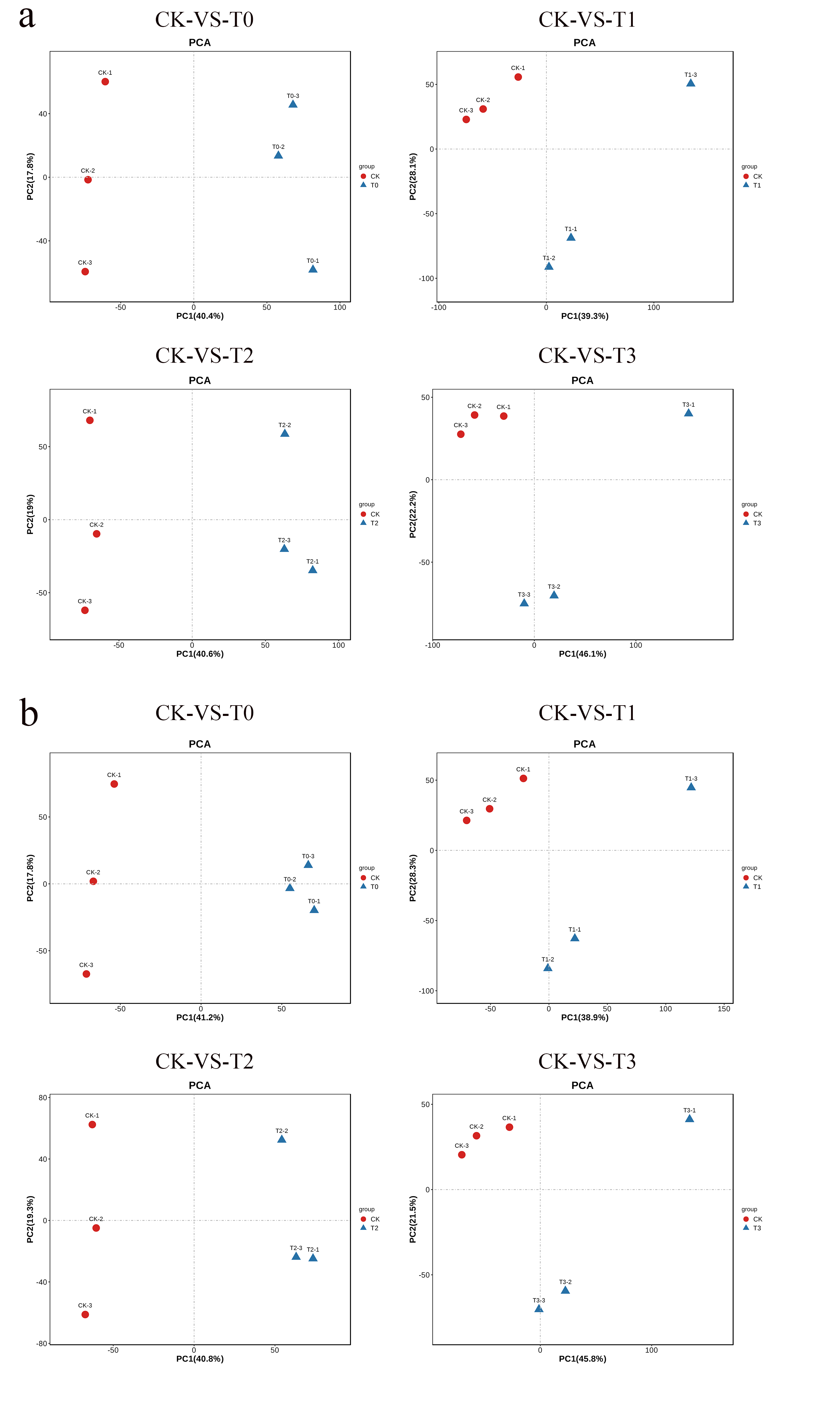


**Figure S3**


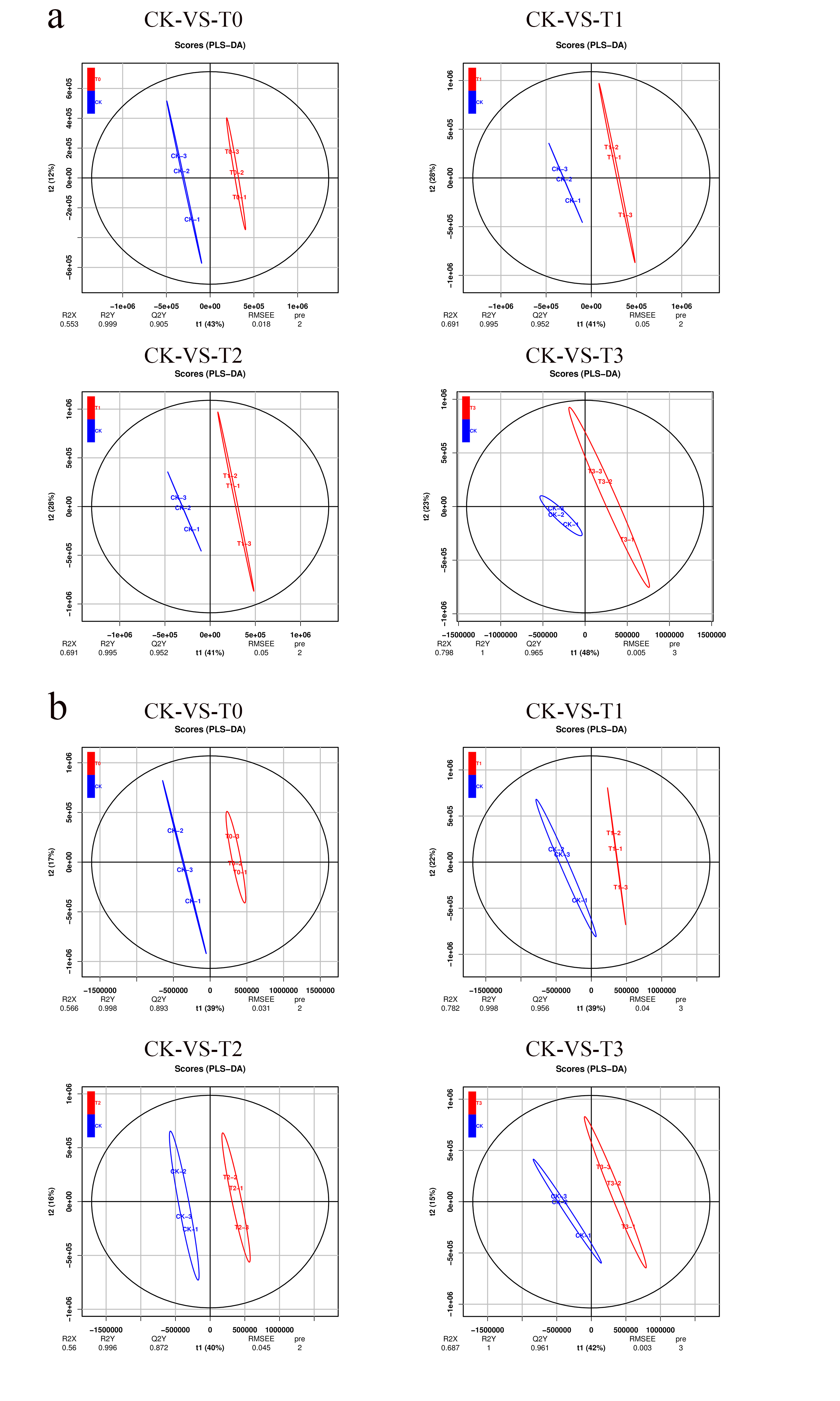


**Figure S4**

**
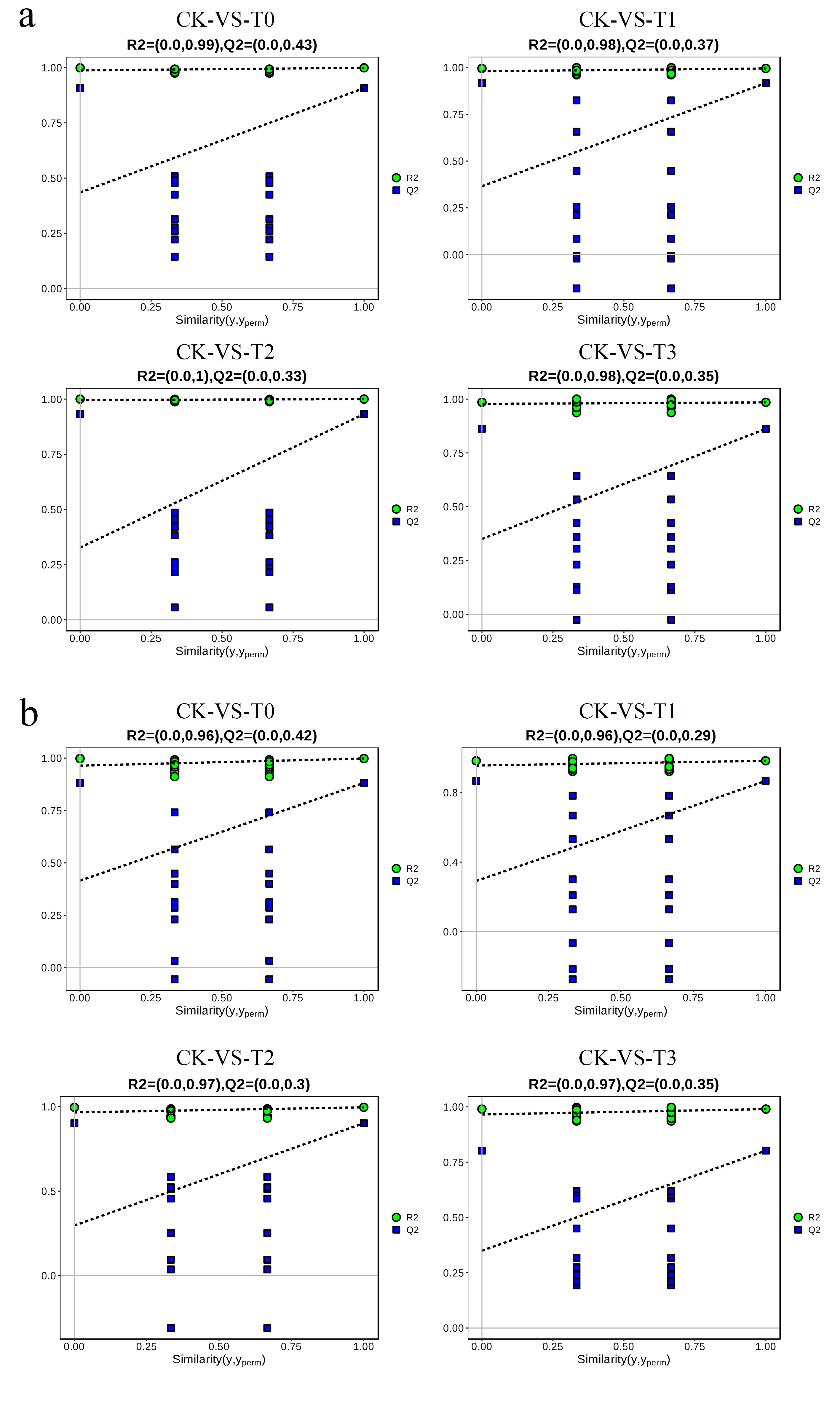
**

**Figure S5**


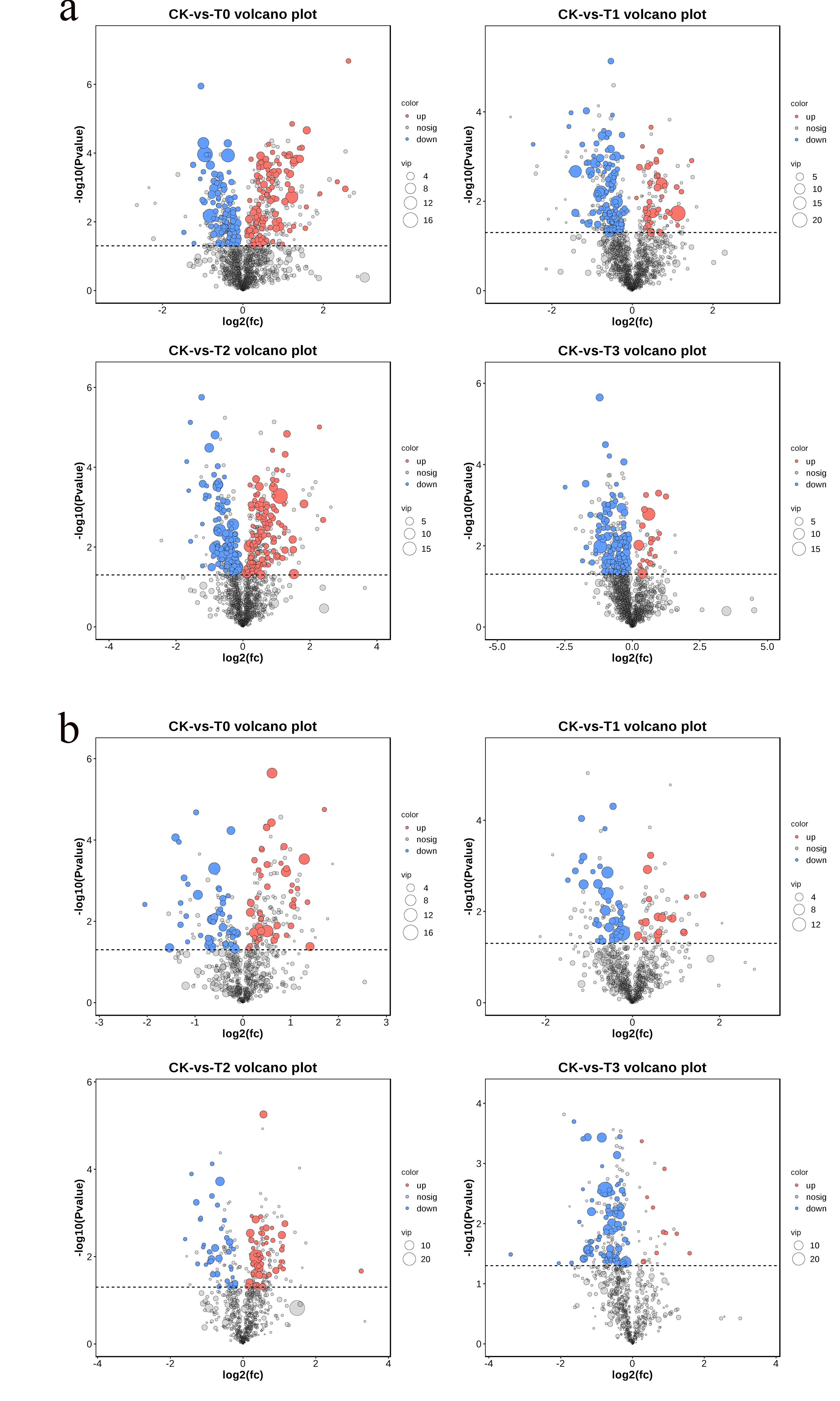


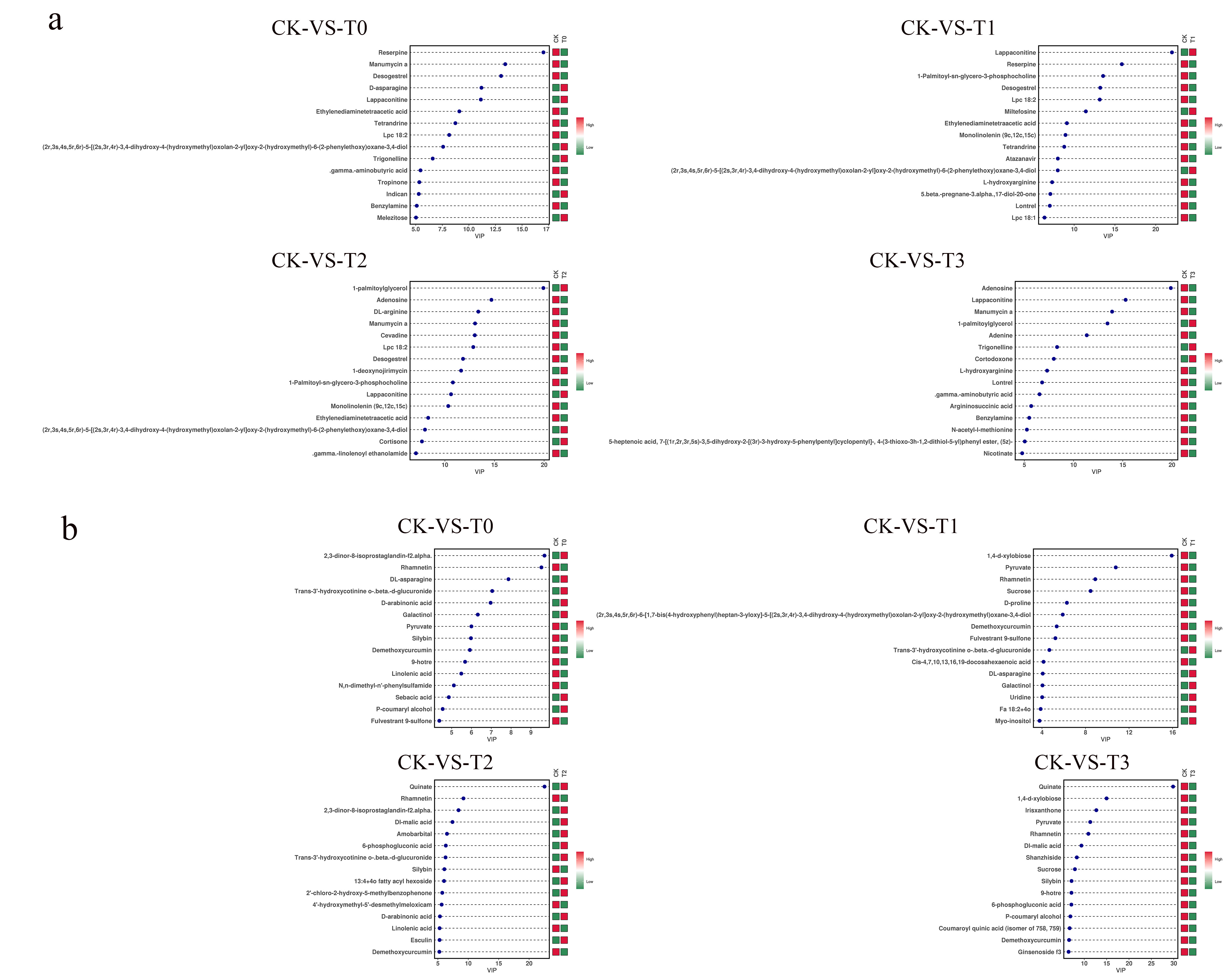
Figure S6
